# Flood occurrence analysis in small urban catchments in the context of regional variability

**DOI:** 10.1371/journal.pone.0276312

**Published:** 2022-11-03

**Authors:** Bartosz Szeląg, Roman Suligowski, Grzegorz Łagód, Ewa Łazuka, Paweł Wlaź, David Stránský, Francesco De Paola, Francesco Fatone

**Affiliations:** 1 Department of Geotechnics and Water Engineering, Kielce University of Technology, Kielce, Poland; 2 Department of Environmental Research and Geo-Information, Jan Kochanowski University, Kielce, Poland; 3 Department of Water Supply and Wastewater Disposal, Lublin University of Technology, Lublin, Poland; 4 Department of Applied Mathematics, Lublin University of Technology, Lublin, Poland; 5 Department of Sanitary and Ecological Engineering, Czech Technical University in Prague, Prague, Czechia; 6 Department of Civil, Building and Environmental Engineering, University of Naples Federico II, Naples, Italy; 7 Department of Science and Engineering of Materials, Environment and Urban Planning-SIMAU, Marche Polytechnic University, Ancona, Italy; University College Cork National University of Ireland, IRELAND

## Abstract

An original method for analyzing the influence of the meteorological, as well as physical-geographical conditions on the flooding of stormwater in small urban catchment areas is proposed. A logistical regression model is employed for the identification of the flooding events. The elaborated model enables to simulate the stormwater flooding in a single rainfall event, on the basis of the rainfall depth, duration, imperviousness of the catchment and its spatial distribution within the analyzed area, as well as the density of the stormwater network. The rainfall events are predicted considering the regional convective rainfall model for 32 rain gauges located in Poland, based on 44 years of rainfall data. In the study, empirical models are obtained to calculate the rainfall duration conditioning the flooding of stormwater in a small urban catchment area depending on the characteristics of the examined urban basins. The empirical models enabling to control the urbanization process of catchment areas, accounting for the local rainfall and meteorological characteristics are provided. The paper proposes a methodology for the identification of the areas especially sensitive to stormwater flooding in small urban catchment areas depending to the country scale. By employing the presented methodology, the regions with most sensitive urban catchments are identified. On this basis, a ranking of towns and cities is determined from the most sensitive to flooding in small urban catchment areas to the regions where the risk of flooding is lower. Using the method developed in the paper, maximum impervious catchment area are determined for the selected regions of the country, the exceedance of which determines the occurrence of stormwater flooding.

## Introduction

Rapid increases in the urbanization of catchment areas and ongoing climatic changes lead to increased quantities of stormwater runoff and pollutants [1–3]. These factors also contribute to storm overflow, the flooding of manholes and increases in pollutant loads introduced directly to receivers [4, 5]. Additionally, they lead to an increase in the number and frequency of floods in urban catchment areas. Moreover, the frequency of floods is an important parameter describing the operation of stormwater networks regulated in the guidelines for designing sewer systems [6]. Taking into account the complex physics and local character of stormwater flooding, small urbanized catchments covering roads and selected homogenous basins consisting of areas of similar land management have been analyzed in many papers [7–9]. Under these conditions, the timing of catchment reactions to rainfall events is minimized. In effect, such a solution enables a limit to the influence of the selected factors on the analyzed phenomenon and allows the determination of the selected relations.

During a flood in an urban area, stormwater is spilled on the surface of the basin, which determines economic losses, health risks, and infrastructure damage. During stormwater flooding, traffic is hindered as well, leading to possible vehicle damages, and generating the social losses connected with extended travel time. Therefore, it is necessary to conduct flood modeling aiming at simultaneously controlling and reducing the occurrence of stormwater flooding in urban catchments. At present, rainfall-runoff models are usually used for this purpose [10, 11]. The development of such models requires collecting data on land development and stormwater networks, as well as rainfall and hydrologic data containing the information on the variability of flows. Among others, the Storm Water Management Model (SWMM) is one of the most commonly employed solutions for this purpose and is also used for simulating stormwater flooding events [12]. Since the issue of stormwater floods in catchment areas is a current topic resulting from the need to modernize existing drainage networks (Low Impact Development–LID, canal retention, etc.), numerous modifications of mathematical models have been proposed to simulate the course of stormwater floods over time [8, 11, 13].

Due to the complexity associated with the requirements of collecting detailed information on the studied urban drainage system (e.g., the need to conduct uncertainty analysis, data collection costs, etc.) and collecting reliable measurement data to predict stormwater flooding, available models can only be applied locally to a single catchment area. Taking into account the above-mentioned limitations, statistical models have also been employed for stormwater flood modeling [14, 15]. However, similar to physical models, statistical models have the limitation of being local and cannot be then applied to other catchments. Moreover, in the abovementioned papers, the rainfall characteristics were covered to a limited extent, and some aspects related essentially to the influence of local rainfall characteristics or of the location of the considered urban catchment on stormwater flooding (i.e., at a country or province scale, etc.) were not investigated in detail. The papers by Guo and Quader [16] and De Paola and Ranucci [17] confirm that the results of spatial analysis play a key role in designing and dimensioning the technical infrastructure (i.e., LID systems, including detention tanks). This is important for the estimated, quick selection of devices, which represent very relevant issues at the stages of the conceptualization and expansion of urban areas when considering the sustainable development principles.

Thus far, the intensity of rainfall events that contribute to flooding in small urban catchments in terms of area has not been determined. This factor is essential for making decisions related to the sustainable development of urban areas aimed at minimizing the influence of rainfall events on the efficiency of stormwater drainage networks [18, 19]. Understanding this factor is important for the long-term development of urban catchment areas and the selection of appropriate solutions, and is essential for adopting correct strategies to properly shape various urban catchment areas. This is of key importance for the appropriate selection of solutions consisting of systems enabling to achieve the required hydraulic (minimizing the number of flooding events and the quantities and volumes of storm overflows) and qualitative effects (reducing the amount of pollutants introduced into the receiver).

Nevertheless, the basic quantitative rainfall description form involves the models of the dependence of rainfall depth or intensity on the duration and probability of exceedance of the rainfall event. These relationships, developed for numerous geographical regions of the world, are qualitatively comparable and usually presented in the form of DDF (depth-duration-frequency) or IDF (intensity-duration-frequency) curves for various rainfall exceedance probabilities [20, 21]. In the case of Poland, the most popular and up-to-date rainfall formula used for any location in the country (except for in the mountainous or sub-mountainous areas) is the probabilistic model developed by Bogdanowicz and Stachy [22]. However, using the so-called local rainfall formulas, i.e., the relations between the abovementioned characteristics (maximum rainfall depth, rainfall duration) based on the rainfall observations in the considered area, determined individually for a given city, and thus reflecting the local rainfall specificity in the best possible way, is a superior solution. An example of such a formula that is currently gaining popularity is the PANDa (Polish Rainfall Intensity Atlas) local rainfall model [23], which was modeled on the KOSTRA atlas in Germany [24] and constitutes a tool aiding in the design of stormwater and drainage systems in Polish cities. The analyses reported in numerous papers have shown the high influence of rainfall origin on rainfall dynamics [25–27], as highlighted by García-Bartual and Andrés-Doménech [28], who determined the IDF curves for convective rainfall events in the Valencia region (Spain).

Rainfall is universally classified into three types [29, 30] convective, cyclonic and orographic. Only real-time rain events of convective origin are distinguished in the manuscript. The main distinguishing feature between convective precipitation in an air mass and frontal precipitation in mid-latitudes is its spatial extent and duration. The range of convective precipitation associated with local air circulation is much smaller than in the case of travelling extratropical cyclones with weather fronts. Convective precipitation induced by single thunderstorm cells, their complexes or squall lines is short-lived, but it is characterized by high average intensity [31] and causes flash floods [32, 33]. On the other hand, the lifespan of the mechanisms of creating cyclonic precipitation is much longer than that of convective precipitation–on the order of days rather than hours.

The convective rainfall series were separated on the basis of an arduous analysis of the maximum rainfall origin in Poland, carried out at the turn of the 19th and 20th centuries [34, 35]. It was based on meteorological and synoptic analysis in the periods of heavy rainfall events (depth, intensity) in a given area. On the basis of the surface synoptic charts of Europe, published in the Daily Meteorological Bulletin of the Institute of Meteorology and Water Management–IMGW in Warsaw) and a calendar describing the types of atmospheric circulation together with air masses and air fronts [36]. A similar approach is used in the literature [37–39].

The performed calculations based on hydrodynamic models of urbanized and agricultural catchments [40–42] showed that convective rainfall events (short duration and high rainfall intensity) strongly affected drainage systems: flooding of manholes, reservoir overflows, stormwater overflows, etc. Thus, to limit these problems, proper decision-making during the control (or even the modernization) of existing stormwater networks is required. For this reason, the studies concerning the frequency, time-related variability and dynamics of convective rainfall events while taking into account the local climatic conditions of global warming are being performed [43, 44].

The main goal of the present study was to develop an original method for analyzing the influence of local meteorological as well as physical-geographic conditions on stormwater flooding in small urban catchments in Poland. A logistic regression model, also known as a binary logit model, based on measurement data from four urbanized catchments, was applied to model flooding in small urban catchments. The logit models developed thus far in the context of flooding are limited to modeling the flooding from individual manholes and do not thoroughly consider the catchment characteristics in the context of stormwater floodings. On the other hand, the existing tools for catchment flooding identification in terms of large areas [45, 46] are complex and difficult to implement. Therefore, there is a need to develop simple tools for flooding identification, which will allow their use in the catchment management phase and enable catchment management in terms of large area as well as the influence of local development conditions, including land use plans. The catchment area is the basic unit of an urban catchment in terms of which, the sewer network performance can be assessed in the context of legal regulations [6].

In comparison to other models (such as linear discriminant models, regression trees, artificial neural networks and their modifications, support vectors), the advantage of using a logistic regression model is that its simulation result constitutes the value of a probability. This enables the analysis of the selected independent variables on the occurrence of the modeled phenomenon, stormwater flooding in this case. The logit model has been applied in the analysis of a drainage network operation for modeling storm overflows [47] as well as for modeling stormwater flooding itself [15]. However, concerning the flooding events, the determined binary models were local, and their application was limited. The solution adopted in this work is aimed at eliminating these limitations. The rainfall events were predicted using a regional convective rainfall model that was determined based on the data from 32 rainfall stations located across the country. Determination of the rainfall event durations that led to stormwater flooding in the small urban catchments, as well as the physical-geographic characteristics of the catchments, and the local characteristics of rainfall events was then performed. The assumed method of calculations allowed the control of the catchment urbanization process accounting for the local rainfall, and meteorological characteristics were developed. By applying these models, it is possible to determine the maximum impervious area of a given catchment in which flooding occurs, considering the local rainfall characteristics. A methodology enabling the identification of the areas that are especially vulnerable to stormwater flooding in small urban catchments in relation to the country scale was proposed.

## Methods and data

### General description

**Fig 1:**
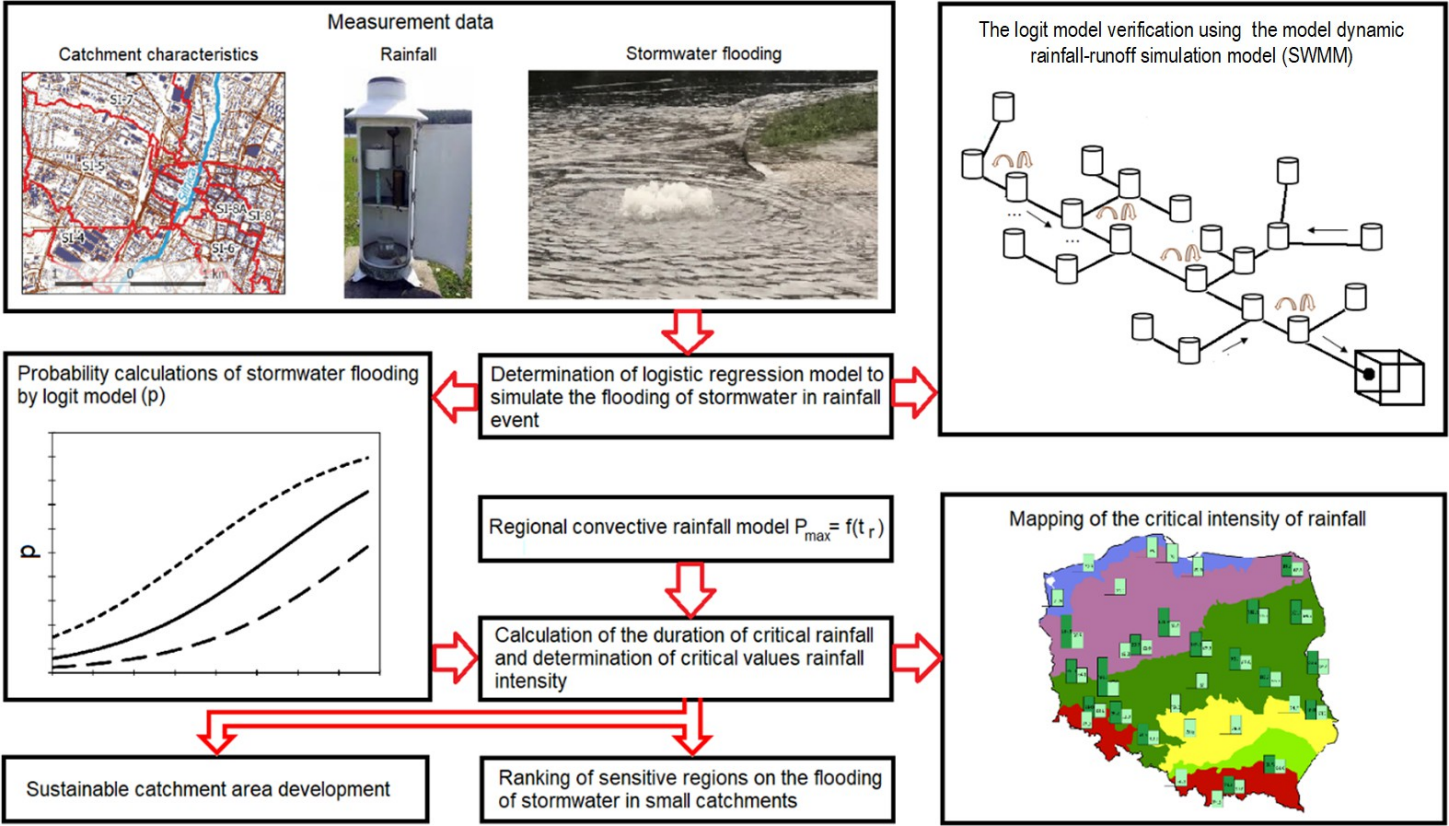
Calculation algorithm of the model used for analyzing the influences of the local catchment and rainfall characteristics on flooding in a small urban catchment.

A novel application of a logistic regression model for the simulation of stormwater flooding in small urban catchments was proposed (see Fig 1).

Regional convection rainfall models were determined for Poland (Central Europe): P_max_ = f(tr), where P_max_ is maximum rainfall depth designated from a time series and t_r_ is rainfall duration. By employing the developed logit model, the empirical dependences necessary for the calculation of the rainfall duration leading to stormwater flooding in a small urban catchment were determined, accounting for the physical and geographical characteristics of the given catchment. Thus, the minimal values of rainfall intensity resulting in stormwater flooding in urbanized catchments in different parts of Poland were determined using the P_max_ = f(t_r_) curves. The dependences enabling the analysis of the influence of urban catchment properties, as well as the local rainfall and geographical characteristics, on stormwater flooding were specified as well. Thus, the adopted solution enables the implementation of sustainable development both locally and globally. Moreover, the adopted solution allows identifying the sensitivity of regions to stormwater flooding in urban areas. This solution is simplified compared to the classic solution, in which flooding is usually identified through a rigorous hydrodynamic model.

Rainfall-runoff modeling is very much a research focus today, especially regarding micro-scale modeling, dual-drainage models, the interaction between groundwater and surface flow, etc. However, this modeling needs definitely much more data, as well as economic and time efforts than the proposed method. Moreover, the proposed flooding model is universal and can be applied to other urban catchments. In contrast to the topic of previous articles [14, 15], the methodology proposed in the present study enables the analysis of flooding phenomena globally (in terms of urban areas in Poland), not only at the local scale, and can also be transferred to other countries in the region (Slovakia, Czechia, Denmark, etc.). In detail, Fig 1 shows the scheme of the calculation algorithm implemented for the simulation and analysis of stormwater flooding. Determination of the model used for stormwater flooding analysis involves the following steps:

- creating regional rainfall models used to calculate a mean of convective rainfall depth as a function of its duration,
- developing a logistic regression model based on measured data (rainfall events, catchment characteristics) to simulate the flooding of stormwater in rainfall event; model validation,
- conducting a sensitivity analysis aimed at determining the influence of the selected variable (area of the catchment, impervious area of the catchment, length of the main channel in the catchment, maximum difference of the land coordinates in the catchment–height difference, length of the main channel, imperviousness of the downstream area, unitary length of the main channel in the catchment per impervious area) on the modeled phenomenon,
- verifying the logistic regression model using a dynamic rainfall-runoff simulation model
- calculating the probability of stormwater flooding for the characteristics of the assumed urban catchment based on the determined regional convection rainfall models P_max_ = f(t_r_),
- calculating the critical rainfall duration (t_cr_) and critical mean rainfall intensity (i_cr_) by describing the stormwater flooding event in an urban catchment area based on the regional convective rainfall models P_max_ = f(t_r_),
- determining the possibilities for catchment urbanization of the selected regions based on the P_max_ = f(t_r_) relationship,
- determining an empirical relation for the identification of the critical rainfall intensity (i_cr_) of the analyzed area.

Following the algorithm described in Fig 1, the following chapters present the next steps in determining the model used for flooding analyses.

### Study area

**Fig 2:**
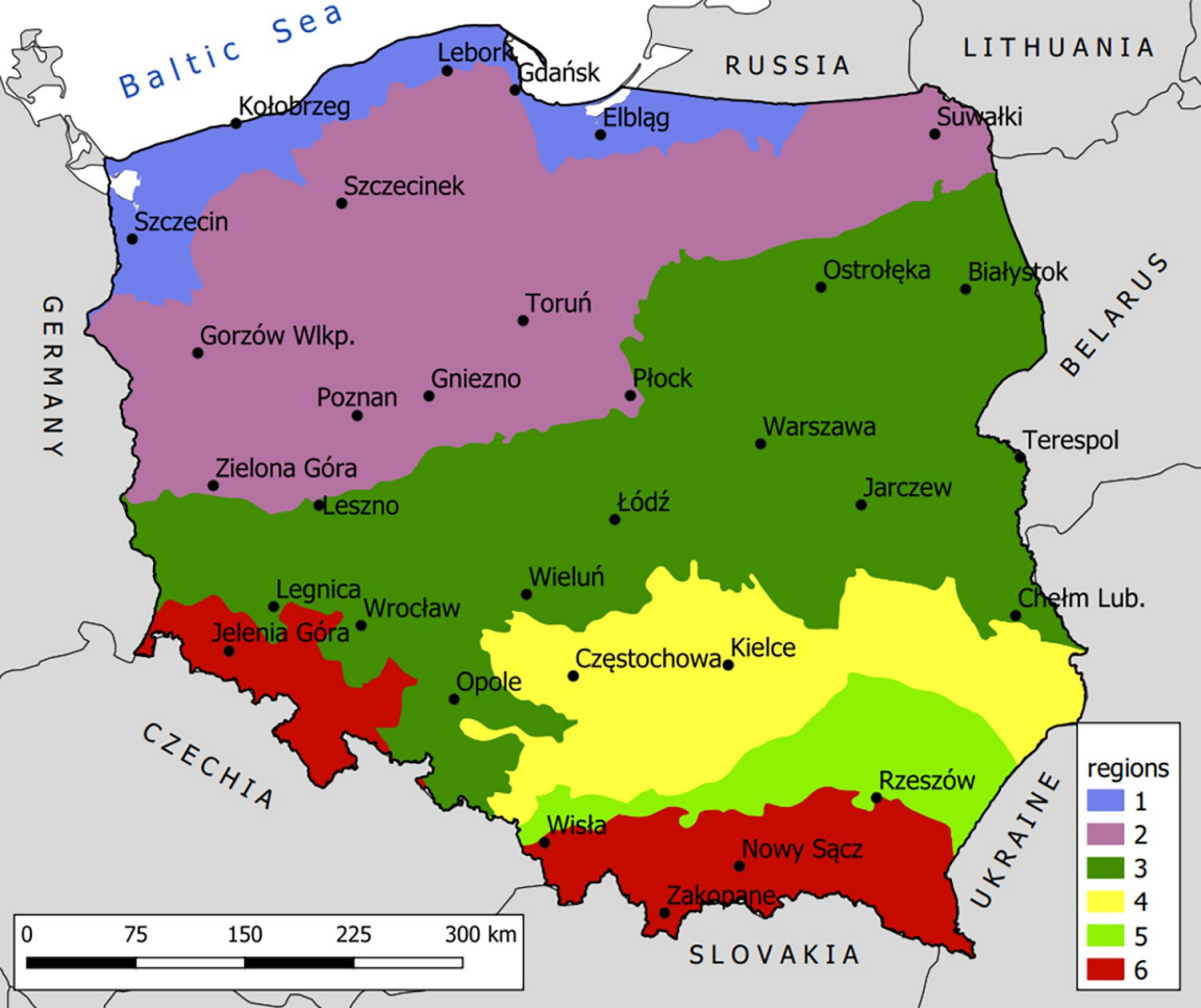
Location of the 32 rain gauges employed in the present study, corresponding to Polish landforms (1 –Baltic coastal lowlands, 2 –lakelands, 3 –central lowlands, 4 –uplands, 5 –sub-mountain basins, 6 –mountains).

To develop the correct rainfall characteristics required for engineering modeling, it is necessary to conduct an analysis of rainfall variability recorded at the largest possible number of measurement points to assess the degree to which the regional rainfall variability influences the relation between the basic rainfall characteristics: the rainfall depth/intensity and its duration. This work involved investigating 32 cities in Poland (Central Europe, over 300 thousand km^2^) by employing the meteorological stations belonging to the Polish Institute of Meteorology and Water Management, National Research Institute (Polish acronym: IMGW-PIB) network. The stations were selected to represent different physical-geographic conditions. The analyzed stations are located in six main regions of Poland (Fig 2).

The most numerous group comprises the stations corresponding to the central lowlands (12) and lakelands (8). The mean distance between stations is approximately 100 km, and the greatest distance is approximately 660 km (Chełm Lubelski–Szczecin). The lowest rainfall measurement point is found at 1 m a.s.l. (Szczecin), whereas the highest is found at 857 m a.s.l. (Zakopane). The Polish climate is defined as temperate and transitional between the warm, maritime climate of western Europe and the continental climate covering Ukraine and Russia. Air masses with different thermal and humidity properties are frequent, contributing to the great differentiation of rainfall types and depths [48].

Rainfall exhibits significant dependence on the topography. The mean annual rainfall depth in Poland (1981−2010) is approximately 600 mm, ranging from approximately 520 mm in the central part (Gniezno, Płock) to almost 700 mm at the Baltic coast (Elbląg) and over 1000 mm in the mountains (Wisła, Zakopane). At most stations located within the upland belt and sub-mountain basins, the mean annual rainfall exceeds 600 mm. The highest rainfall depths occur in the summer months, being 2-3-fold higher than those occurring in winter. The quotient of the warm (V−X) and cold (XI−IV) halves of the year increases southward, ranging from 1.4 (Baltic coast) to 2.2 (mountains), which is a sign of the continental features of the pluvial regime [49]. The rains with the highest intensities occur from May to August, with the greatest frequency in July [50]. The basic information about the geographical location of the rainfall measurement points is presented in S1 Table.

### Rainfall data

When dealing with small urban catchments, from the point of view of modeling stormwater flooding in a rainfall event, it is important to account for short rainfall events with high intensities. These events are mainly connected with convection in air masses [35, 39] Their characteristic features in mid-latitudes are a short range (up to 10 km^2^) and high mean intensity. In Poland, these events last up to 90 min [35], and in cities, in addition to stormwater overflows [47], they also cause flash floods [51, 52].

The calculation procedures were conducted by considering historical rainfall events with real durations up to 90 min (convective in an air mass) occurring in the six warm months (V−X) in the 1961−2005 period based on analog records obtained by using a traditional float pluviograph. Thus, the total depth and the mean intensity of rainfall were analyzed. The rainfall that may last longer (frontal, in a convergence zone) was not the subject of the analysis, although locally it may cause large peak-storm intensity. The collected data are homogenous, both concerning the measurement method and the time step used for recording the rainfall depth at each station [53]. The dry period duration used to distinguish between separate rainfall events was assumed to be four hours [54]. In detail, the obtained database contains the information on the date of each rainfall event and its total depth, duration, and mean intensity.

The frequency of rainfall events lasting up to 90 min with depths over 0.2 mm is characterized by high variability, depending on the locations of the meteorological stations. In the analyzed multiannual period, there were 1,728−2,951 rainfall events in the summer half-year period, which amounts to an annual mean of 57 to 98 events. The highest frequencies of events occurred in northern Poland (Białystok, Szczecin, and Kołobrzeg) and the mountains (Zakopane and Jelenia Góra), although these events were usually not very intense (<0.6 mm). Over 50% of the total convective rainfall (in air mass) corresponded to the events lasting up to 30 min, and their number decreased with increasing duration at all stations. The total depth of convective rainfall is low, but due to the short duration of these events, the intensity is very high, greatly exceeding 1 mm min^-1^.

### Regional model of convective rainfall

For the purpose of the conducted analysis, the designated rainfall events were allocated to one of five proposed time intervals: 1−10 min, 11−20 min, 21−30 min, 31−60 min, and 61−90 min. Their timespans were adjusted to the decreasing frequency of rainfall events to ensure at least several rainfall events in each year of the multiannual period. Within each dataset for all measurement points, the 45 highest rainfall event values were selected, regardless of the year in which they occurred [55], which can be considered a special case of designating the series of maximum values with the peak-over-threshold method [56]. For each rainfall station, an optimal connection consisting of the rainfall depth (P) event as a function of its duration (t_r_) was determined with a second-degree polynomial function:

$$P_{max}(t_{r})=a_{1}\cdot t_{r}^{2}+a_{2}\cdot t_{r}+a_{0}$$ (1)

This solution was previously employed in the papers by Kupczyk and Suligowski [53] and Suligowski [57]. The values of the coefficients a_1_, a_2,_ and a_0_ are summarized in S1 Table.

**Fig 3:**
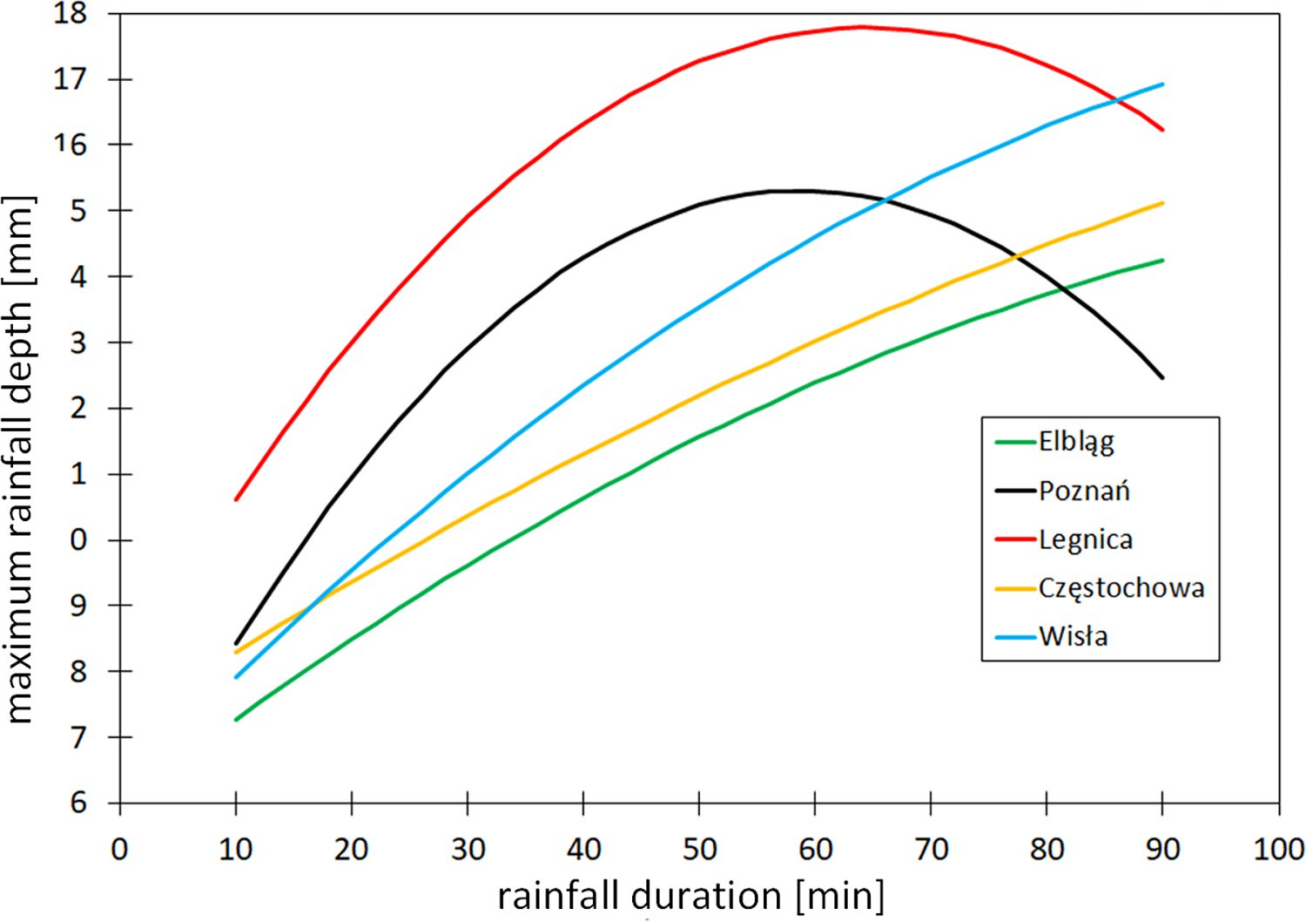
Mean high of convective rainfall depths as a function of their duration at selected meteorological stations (names in Fig 1) located in different regions of Poland.

The conducted analyses indicated that the mean of the high convective rainfall in an air mass are characterized by high variability (Fig 3). At all considered stations, the mean of high observed depth increases along with the rainfall duration, up to approximately 60 min. This tendency is sustained in the case of longer events (61–90 min) only at the measurement stations located in the areas with different reliefs (mountains–Wisła, uplands–Częstochowa) and those adjacent to the Baltic Sea (Elbląg). At the majority of the measurement stations located in lowlands (Poznań, Legnica), there is a clear tendency towards decreasing the mean of high rainfall depth in dependence of the duration interval compared to the shorter events.

The greatest increase in the mean of rainfall depth lasting up to 60 min (coefficient values of a_1_ < 2.8∙10^−3^ and a_2_ > 0.31 –S1 Table) occurs at the stations in central Poland: Jarczew, Leszno, Łódź, and Warsaw (central lowlands), Gniezno, Poznań, and Suwałki (lakelands) and in the southeast (Nowy Sącz and, Rzeszów). In turn, the most balanced rainfall, characterized by the lowest maximum efficiency in all short time intervals (1–10, 11–20, 21–30, 31–60 min), occurs in Kielce, Częstochowa (uplands), and Wieluń (a_1_ *=* 3.0∙10^−4^ and a_2_ = 0.11), as well as in Elbląg and Kołobrzeg (a_1_ *=* 5.0∙10^−4^ and a_2_ = 0.137) on the Baltic coast. The characteristic feature corresponds to the highest depth of convective rainfall events with a duration of 61–90 min in the stations located in the areas with different reliefs: mountains (Zakopane and Nowy Sącz) and the terminal moraine ridges of the Pomeranian Lakeland (Szczecinek).

In these regions, the occurrence of convective rainfall is also influenced by topographical conditions and local dynamic factors, which are sometimes important in the formation of convective clouds (forced convection); this is confirmed by the climate of these areas [58]. The rainfall depths in these cities exceed 17 mm and are much larger, in a similar period than the rainfall depths in the large urban agglomerations located in lowlands (Białystok– 9.3 mm, Poznań– 12.3 mm, Łódź– 12.8 mm). This discrepancy can be influenced by complex thermal and dynamic processes connected with the urban heat island effect [59, 60] and urban aerosols that modify the physical properties of condensation nuclei [61]. Similar rainfall depths were observed in the cities located near the Baltic Sea (Szczecin– 11.0 mm, Gdańsk– 11.2 mm), where the evaporative cooling effect of the open water surface leads to the inhibition of convection [62].

### Catchment characteristics

**Fig 4:**
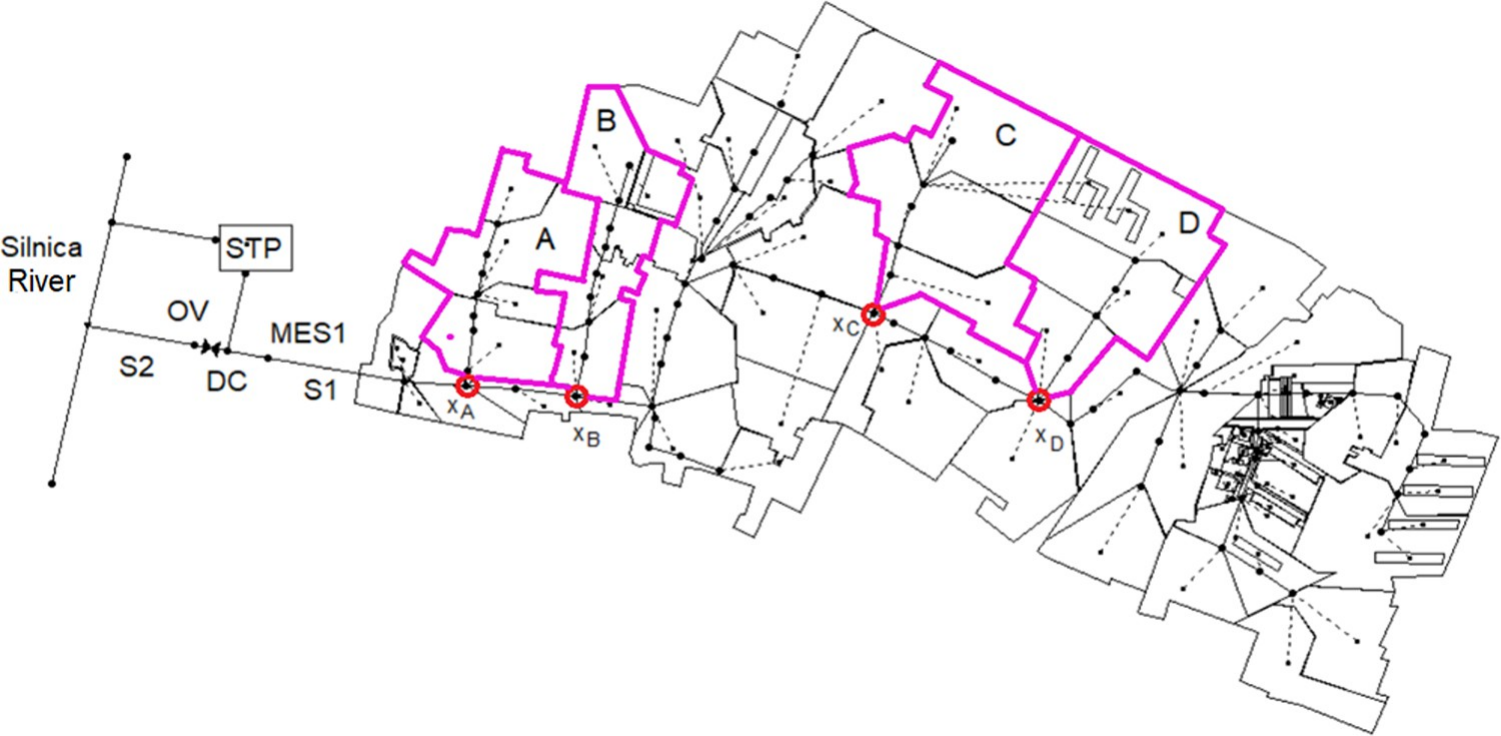
Location of partial catchments A, B, C, and D in the Si9 urban catchment.

The analysis pertaining to the determination of the logistic regression model for simulating the flooding of stormwater in rainfall events was based on four catchments (A, B, C, and D) located in Kielce within the Si9 catchment, having an area of 63 ha (Fig 4). Kielce, the capital city of the Świętokrzyskie Province, is located in central Poland and is served by a separate stormwater system. The analyzed region is inhabited by 21.4 people ha^-1^. Stormwater is discharged from the considered catchment to the Silnica River via the S1 collector. Prior to discharge, river stormwater is treated at Q_m_ = 200 L s^-1^. The annual rainfall depth in the catchment amounted to 537–757 mm, whereas the number of days with rainfall varied in the range of 155–266. The mean annual air temperature ranged from 8.1°C to 9.6°C, and the number of days with snowfall amounted to 36–84.

A permanent monitoring of the stormwater quantity from the catchment indicated that, during dry weather, the inlet flow rate varied from 1 to 11 L s^-1^.

The selection of partial catchments to be used for analyses was governed by multiannual observations about the effect of urbanization on the operation of the stormwater system in the period of 1980–2006 in the center of Kielce, which was previously described by Ciupa [63]. These observations indicated that stormwater flooding occurred in the areas of the considered catchments (A, B, C, and D) following intensive rainfall events. This was also confirmed by permanent monitoring carried out by the stormwater network manager in Kielce. The selection of catchments to be used for analyses was dictated by their physical and geographic spatial distributions, as well as by the possibility of observing the operation of the stormwater network during rainfall events. The physical and geographical characteristics of the urbanized catchment that might influence the surface water flood risk include, i.e.: catchment area, impervious area of catchment, maximum height difference of the land coordinates in the catchment. The basic characteristics of the analyzed catchments are presented in Table 1. Detailed information concerning the analyzed catchment was provided by Kiczko et al. [64].

**Table 1:** Physical and geographic characteristics of the A, B, C, and D catchments.

Catchment	F	Imp	L_tot_	dH	L_k_	Imp_b_	G_k_
	(ha)	(-)	(m)	(m)	(m)	(-)	(m ha^-1^)
A	8.066	0.306	192.2	1.62	147	0.089	0.042
B	5.447	0.512	303.5	3.38	478	0.151	0.011
C	6.157	0.452	170.0	1.91	1626	0.408	0.036
D	7.020	0.487	215.3	1.27	1850	0.512	0.033

where F–catchment area, Imp–impervious area of catchment, L_tot_−length of the main collector in the catchment, dH–maximum height difference of the land coordinates in the catchment, L_k_−length of the collector measured from point x_A, B, C, D_ to the diversion chamber (DC), Imp_b_−imperviousness of the area located below the analyzed catchment (point x_A_, x_B_, x_C_, x_D_), and G_k_−density of the stormwater network defined as the length of the main collector via the impervious surface divided by the catchment area.

The measurements of stormwater flow rate and pipe filling depth were conducted using a MES1 flow meter located 4.0 m before the DC as part of the monitoring (2008–2018); the flow rate values were recorded with a 1-min resolution. Field studies of stormwater flooding in the A, B, C, and D catchments were also conducted in the 2009–2015 period [65]. This measurement period was assumed, because a significant increase in the imperviousness of the area has occurred since 2016.

As part of the conducted monitoring, it was possible to observe that stormwater flooding led to local flooding and hindered traffic on roads. Identification of rainfall events during the conducted monitoring of stormwater flooding was conducted based on short- and long-term forecasts by the Polish Institute of Meteorology and Water Management, available on the *www.pogodynka.pl* and *www.meteo.pl* websites. The main benefit of the adopted model was the possibility to obtain the information on the rainfall event dynamics on a qualitative scale (light, strong, moderate rain) in advance. During the field studies in 2009–2015, it was shown that 90 rainfall events took place in the period 2009–2015: four flooding events occurred in catchment A, seven flooding events in catchment B, eight flooding events in catchment C, and three flooding events in catchment D [65]. In this study, the flooding events were identified as rainfall, during which the depth of the water layer within the road lane was not lesser than the curb height, which corresponded to approx. 0.1 m.

### Logistic regression model

#### Stormwater flooding in a rainfall event

On the basis of literature data [15, 66, 67], the probability of stormwater flooding in a rainfall event (p) can be described with the following dependence:

$$p=\frac{exp(\alpha_{0}+\alpha_{1}\cdot P_{tot}+\alpha_{2}\cdot t_{r}+\alpha_{3}\cdot Imp+\cdots +\alpha_{i+3}\cdot x_{i+3}(Gk,{Imp}_{b}))}{1+exp(\alpha_{0}+\alpha_{1}\cdot P_{tot}+\alpha_{2}\cdot t_{r}+\alpha_{3}\cdot Imp+\cdots +\alpha_{i+3}\cdot x_{i+3}(Gk,{Imp}_{b},))}$$ (2)

where α_0_, α_1_, α_2_, α_3_, and α_i+3_ are empirical coefficients determined with the maximum likelihood estimation method (to identify the coefficients in the logistic regression model, the forward stepwise method was used to eliminate the correlation of independent and reference variables to models as analyzed in the paper); i–the number of catchment characteristics included in the model, where: i = 1, 2, 3, …j; j–number of independent variables describing the catchment characteristics included in the Table 1; Ptot−total rainfall depth;

On the basis of the literature data reported by Jato-Espino et al. [14], in the analyses, it was assumed that stormwater flooding during a rainfall event occurs when p ≥ 0.5, which corresponds to the following dependence:

$$\alpha_{0}+\alpha_{1}\cdot P_{tot}+\alpha_{2}\cdot t_{r}+\alpha_{3}\cdot Imp+\ldots +\alpha_{i+3}\cdot x_{i+3}(Gk,{Imp}_{b})\geq 0$$ (3)

Thus, the determined logit model Eq (2) and the abovementioned dependence Eq (3) enable an analysis of the influence of selected catchment characteristics on stormwater flooding, accounting for the local rainfall conditions. This is an important functionality of a logit model and the assumed calculation methodology, since it enables the determination of the sustainable development concept for urban catchments and the method of stormwater management.

An assessment of the predictive capabilities of the logit model was carried out with the two measures of fit [66, 68], namely Sensitivity (SENS) and Specificity (SPEC), which determine the accuracy of the data classification in a set comprising the cases when flooding occurred and when it did not occur, respectively. The logit model was determined using the measurement data from four urban catchments (Fig 3).

#### Minimum rainfall event duration and rainfall intensity

One of the frequently employed criteria for the analysis of stormwater network operations as a function of stormwater flooding phenomena is the minimum (critical) rainfall intensity i_cr_ = P_max_(t_cr_)/t_cr_ (L s^-1^ ha^-1^) of events leading to flooding in the catchment area. The obtained critical rainfall duration (t_cr_) is the maximum value for which stormwater flooding occurs in an urban catchment. By using Eq (1) in Eq (3) for p = 0.50, the following is obtained:

$$\alpha_{0}+\alpha_{1}\cdot (a_{1}\cdot t_{r}^{2}+a_{2}\cdot t_{r}+a_{0})+\alpha_{2}\cdot t_{r}+\alpha_{3}\cdot Imp+\cdots +\alpha_{i+3}\cdot x_{i+3}(Gk,{Imp}_{b})=0$$ (4)

By transforming Eq (4) and introducing the appropriate data, the minimum duration of rainfall events (t_cr_) can be determined from the following formulas:

$$t_{cr}=\frac{-(a_{2}\cdot \alpha_{1}+\alpha_{2})+\sqrt{{\left(a_{2}\cdot \alpha_{1}+\alpha_{2}\right)}^{2}-4\cdot a_{1}\cdot \alpha_{1}\cdot \theta }}{2\cdot a_{1}\cdot \alpha_{1}}$$ (5)

$$\theta =\alpha_{1}\cdot a_{0}+\alpha_{3}\cdot Imp+\cdots +\alpha_{i+3}\cdot x_{i+3}(Gk,{Imp}_{b})$$ (6)

On the basis of Eqs (5) and (6), for a given region described by the P_max_ = f(a_0_, a_1_, a_2_, t_r_) model, the minimum rainfall duration caused by convective rainfall at which stormwater flooding occurs can be calculated. For modeling stormwater flooding in small urban catchments, a minimum rainfall duration of t_cr,min_ = 10 min was assumed. When the value of t_cr_ obtained from Eqs (5) and (6) was shorter than t_cr,min_ = 10 min, then p < 0.5, which denoted no stormwater flooding for the rainfall characteristics P_max_ = f(a_0_, a_1_, a_2_, t_r_). Following the dependences denoted in Eqs (5) and (6), calculations of the critical rainfall event duration leading to stormwater flooding in each catchment with the assumed physical and geographic characteristics for 32 meteorological stations in Poland were carried out. Thus, the maps reflecting the variability of the i_cr_ value were prepared depending on the characteristics of the catchment area and its location in the mesoregion.

#### Ranking of the areas sensitive to stormwater flooding in a small urban catchment considering variable imperviousness

Creating a ranking and comparison of data in the form of multidimensional vectors are complex tasks that are carried out using clustering methods (hierarchical cluster analysis; HCA). Ranking and comparing these forms of data requires the implementation of numerical algorithms, and the interpretation of the results requires specialist knowledge. In this paper, the basis for the creation of a ranking system indicating the areas that are sensitive to stormwater flooding was the matrix $J=[i_{{Imp}_{s,r}}]$, where *s* = 0,1,…,*t*; *r* = 1,2,…,*k*, expressed here in the following form Eq (7):

$$J=[\begin{array}{cccc} i_{Imp_{0,1}} & i_{Imp_{0,2}} & \ldots & i_{Imp_{0,k}}\\ i_{Imp_{1,1}} & i_{Imp_{1,2}} & & i_{Imp_{1,k}}\\ \vdots & \vdots & \ddots & \vdots \\ i_{Imp_{t,1}} & i_{Imp_{t,2}} & \cdots & i_{Imp_{t,k}} \end{array}],$$ (7)

where r = 1, 2, 3, …, k indicates the number of regions (here: rainfall stations) assumed for the stormwater flooding analysis; s = 0, 1, 2, 3, …, t indicates the number of imperviousness variants assumed for calculations so that Imp_1,r_−Imp_0,r_ = Imp_s,r_−Imp_s-1,r_ = Δ = const (in the present manuscript, Δ = 0.01 and the minimum and maximum values of Imp are 0.36 and 0.60, respectively, as appropriate; this resulted in t = 25 variants); and $i_{{Imp}_{s,r}}$ represents the minimum convective rainfall intensity for *r* at the given rainfall station for the given Imp value.

In the analyses, it was assumed that the greater the Imp value for which flooding occurs in *r* is, the lower the sensitivity is in relation to the areas in which flooding occurred for lower Imp values. This stems from the fact that, along with an increase in imperviousness, the duration of a rainfall event at which flooding can occur is extended. As a result, the mean rainfall intensity that causes flooding is decreased, leading to the problems with the operation of the stormwater network.

On the basis of the presented matrix, in the calculations, it was assumed that if $i_{{Imp}_{s,r}}>0$, then, for the input described with the [x_long_ y_lat_ h_alt_ P_a_ Imp = Imp_b_ t_cr_] vector, stormwater flooding occurs in the analyzed region, and the value of the flooding parameter is equal to $\pi_{{Imp}_{s,r}}=1$. Otherwise, i.e., when $i_{{Imp}_{s,r}}=0$, then $\pi_{{Imp}_{s,r}}=0$. For these assumptions, the matrix described by Eq (7) may be recorded as:

$$J^{\prime }=[\begin{array}{cccc} \pi_{Imp_{0,1}} & \pi_{Imp_{0,2}} & \ldots & \pi_{Imp_{0,k}}\\ \pi_{Imp_{1,1}} & \pi_{Imp_{1,2}} & & \pi_{Imp_{1,k}}\\ \vdots & \vdots & \ddots & \vdots \\ \pi_{Imp_{t,1}} & \pi_{Imp_{t,2}} & \cdots & \pi_{Imp_{t,k}} \end{array}].$$ (8)

On the basis of these data, so-called sensitivity indices are calculated for *k* areas using the following formula:

$$p_{t,r}=\frac{\sum_{s=0}^{t}\pi_{{Imp}_{s,r}}}{t+1}$$ (9)

where, as previously, *r* = 1,2,…,*k*. The greater the p_t,r_ value is, the higher the sensitivity of *r*, which indicates the area of stormwater flooding in a small urban catchment. The calculated values of p_r_ for *r* indicate this area and constitute the basis for creating a ranking of areas from the highest sensitivity to flooding (p_r_ → max) to the lowest (p_r_→ min). For example, if, for the calculated *r* of a given rainfall station, the values of $i_{{Imp}_{s,r}}$ are greater than 0 for two or three Imp variants, the sensitivity indices are p_t,r_ = 2/25 = 0.08 and p_t,r_ = 3/25 = 0.12, respectively. Assuming that the value of p_t,r_ = f(Imp) for the analyzed area changes linearly in the range of p_t,r (min)_(Imp_0,r_)– 1.0(Imp_t,r_), the following relation can be written:

$${Imp}_{gr,r}=\frac{p_{t,r}\cdot ({Imp}_{0,r}-{Imp}_{t,r})-p_{t,r(min)}\cdot {Imp}_{0,r}}{1-p_{t,r(min)}},$$ (10)

where p_t,r(min)_−minimum value of the sensitivity index (p_t,r_) for the region considered.

#### Sustainable development of urban catchments in terms of mitigating stormwater flooding

An important functionality of the determined model is the possibility of analyzing the influence of catchment characteristics on stormwater flooding in urban catchments by applying Eq (4). Assuming the lack of flooding in a catchment (p = 0.50) and that its value is determined by its impervious area (Imp), and the urbanization of the areas below the analyzed one (Imp_b_), relationship Eq (4) can be written in the following form:

$$\alpha_{0}+\alpha_{1}\cdot (a_{1}\cdot t_{cr}^{2}+a_{2}\cdot t_{cr}+a_{0})+\alpha_{2}\cdot t_{cr}+\alpha_{3}\cdot Imp+\alpha_{4}\cdot {Imp}_{b}=0$$ (11)

By adapting Eq (11), the values of Imp and Imp_b_ for a selected region in an urban catchment in which stormwater does not cause flooding can be determined:

$$Imp=\frac{1}{\alpha_{3}}\cdot (-\alpha_{0}-\alpha_{1}\cdot (a_{1}\cdot t_{cr}^{2}+a_{2}\cdot t_{cr}+a_{0})-\alpha_{2}\cdot t_{cr}+\alpha_{4}\cdot {Imp}_{b})$$ (12)

$${Imp}_{b}=\frac{1}{\alpha_{4}}\cdot (-\alpha_{0}-\alpha_{1}\cdot (a_{1}\cdot t_{cr}^{2}+a_{2}\cdot t_{cr}+a_{0})-\alpha_{2}\cdot t_{cr}+\alpha_{4}\cdot Imp)$$ (13)

By employing Eqs (12) and (13), the sustainable development of urban areas can be planned. On the basis of Eqs (5), (6), (12), and (13) for 32 rainfall stations characterizing the selected physical-geographic regions of Poland, the areas sensitive to stormwater flooding were identified. The minimum values of imperviousness Imp = Imp_b_, for which stormwater flooding events occurred in the small urban catchments were determined. Thus, a map of the minimum imperviousness, Imp_min_, was created for the analyzed areas of Poland.

#### Verification of the logit model results using the SWMM hydrodynamic model

Verification of the results of the stormwater flooding logit model applied in the investigated catchments was performed using a hydrodynamic catchment model of the Si9 stormwater network created in EPA SWMM 5.0. On the basis of rainfall events and physical-geographic characteristics, a logit model was determined, and flooding in the analyzed catchments was identified, and flooding events calculations in catchments A, B, C, and D, which constitute the partial catchments, were simultaneously performed by using the SWWM hydrodynamic model. The developed catchment model was employed for the identification of stormwater flooding occurrence in the considered partial catchments using the stormwater flooding occurrence criterion. Thus, the results were mainly qualitative.

The model of the Si9 catchment comprises 32 partial catchments, each with an area of 0.12–2.10 ha, and has a total area of 62 ha. The impervious area ranges from 5 to 85%, while the terrain slope amounts to 0.1–4.0%. The retention of the impervious area equals 2.5 mm, whereas the perviousness area equals 6.0 mm. The values of Manning’s hydraulic roughness coefficient for impervious and pervious areas are equal to 0.025 m^-1/3^ s and 0.250 m^-1/3^ s, respectively. The stormwater network comprises 200 nodes (manholes) and 72 sewers (pipelines). Manning’s hydraulic roughness coefficient was assumed to be 0.018 m^-1/3^ s. The model of the catchment was calibrated in relation to the simulation of stormwater runoff using the deterministic and probabilistic methods (GLUE+GSA), which were previously described by Szeląg et al. [69]. The hydraulic model of the urban catchment and storm overflow with the inclusion of stormwater treatment plants was calibrated as well and was described in detail in Szeląg et al. [47]. Jato-Espino et al. [14] and Kotowski et al. [40] discussed the limitations of stormwater flood modeling and presented an approach in which the assessment of stormwater flooding was carried out with a model using high-resolution observations of flow and rainfall. The obtained simulation results may constitute important information for planning further comprehensive field studies on stormwater flooding and aid in making the decisions on the modernization of stormwater networks.

## Results and discussion

### Determination of a logistic regression model for the calculation of stormwater flooding probability

On the basis of stormwater flooding measurements obtained in catchments A, B, and C, as well as rainfall events, the independent variables in the logit model (Eq (2)), which have significant influences on the investigated phenomenon, were determined. Model validation was carried out using the measurement data obtained in catchment D. Thus, the logistic regression equation used to simulate stormwater flooding in small urban catchments was determined to have the following form:

$$p=\frac{exp(0.525\cdot P_{tot}-0.165\cdot t_{r}+10.272\cdot Imp+5.796\cdot {Imp}_{b}+0.024\cdot G_{k}-12.402)}{1+exp(0.525\cdot P_{tot}-0.165\cdot t_{r}+10.272\cdot Imp+5.796\cdot {Imp}_{b}+0.024\cdot G_{k}-12.402)}$$ (14)

where Imp, Imp_b_, G_k_, and t_r_ are as described above; P_tot_−total rainfall depth in a rainfall event calculated from the relationship P_max_ = f(a_0_, a_1_, a_2_, t_r_).

Two basic measures of model accuracy, i.e. sensitivity (SENS) and specificity (SPEC), were estimated. The performed analyses indicated that out of 19 stormwater flooding events that occurred in catchments A, B, and C, a correlation between the calculations and measurements was obtained in 18 cases, which corresponds to SENS = 97.73%. On the basis of the calculations, it was noted that out of 76 rainfall events in catchments A, B, and C, when no road flooding occurred, the simulations matched the measurements in 73 cases, achieving SPEC = 96.05%. In catchment D, when considering the 14 rainfall events when no flooding occurred, the measurements agreed with the calculations in 12 cases, reaching SPEC = 85.71%. Thus, the obtained results indicate a high correlation between the measurements and calculations. The model validation indicated that in eight cases of flooding, the measured results agreed with the calculations in all rainfall events, reaching SENS = 100%. Independent rainfall events measured in catchment areas A, B, C, and D were selected for validation, including two rainfall events each in which either no flooding or flooding occurred.

Considering Eq (14), it can be concluded that, in addition to rainfall characteristics, the characteristics of the catchment area include its spatial distribution in the catchment impervious area and the density of the stormwater system. The abovementioned analysis results are shown in the study of Jato-Espino et al. [14], in which a statistical model for identifying floods in a 10,535 ha catchment area within the city of Espoo (Finland) was developed. Li and Willems [15], in their analysis of two small catchments (Merksem and Duerne in Belgium), showed the impact of stormwater network retention on flooding. They also showed the influence of soil moisture on the analyzed phenomenon, which was possible, because their measurements and flood event identifications were carried out as part of a continuous monitoring regime.

#### Logit model verification using SWMM hydrodynamic simulations

On the basis of the hydrodynamic model of the catchment, in which the analyzed areas (A, B, C, and D) cover the catchments of the side sewers, calculations of stormwater flooding were conducted based on the measurement data of rainfall events. These data were also compared with the results of the calculations performed using logistic regression. A comparison of the calculation results with the measurements is presented in Table 2. In the analysis of the influence of selected characteristics of small urban catchments (spatial imperviousness distribution and density of the stormwater network), the results obtained using a logistic model be reliable. This reliability is also important for the possibility of employing this dependence for other small urban catchments with different physical and geographical characteristics.

**Table 2:** Number of flooding events of logistic regression, SWMM and measurements in catchments A, B, C, and D.

Calculation method	Catchment			
	A	B	C	D
Measurements (flooding)	4	7	8	3
Measurements (no flooding)	26	30	30	14
SWMM (flooding)	3	6	8	3
SWMM (no flooding)	23	29	28	12
Logit (flooding)	3	7	7	3
Logit (no flooding)	24	27	27	13

On the basis of the data reported in Table 2, a high dependence between the measurements and calculations performed with the logit and hydrodynamic models can be seen. The maximum difference between the number of flooding events measured and that modeled using the logit model in the analyzed period is not greater than 1. The maximum difference between the number of events in which no flooding occurred (from measurements) and the number from SWMM modeling is up to 2 events, whereas the difference obtained during field studies and the logit model amounts to 3. Moreover, the calculation results (of flooding or lack thereof) obtained using the logit model and SWMM indicated good agreement. Thus, it can be stated that the determined logit model can be successfully applied for the simulation of the influence of the selected urban catchment characteristics on stormwater flooding.

### Influence of catchment and local rainfall characteristics on stormwater flooding

**Fig 5:**
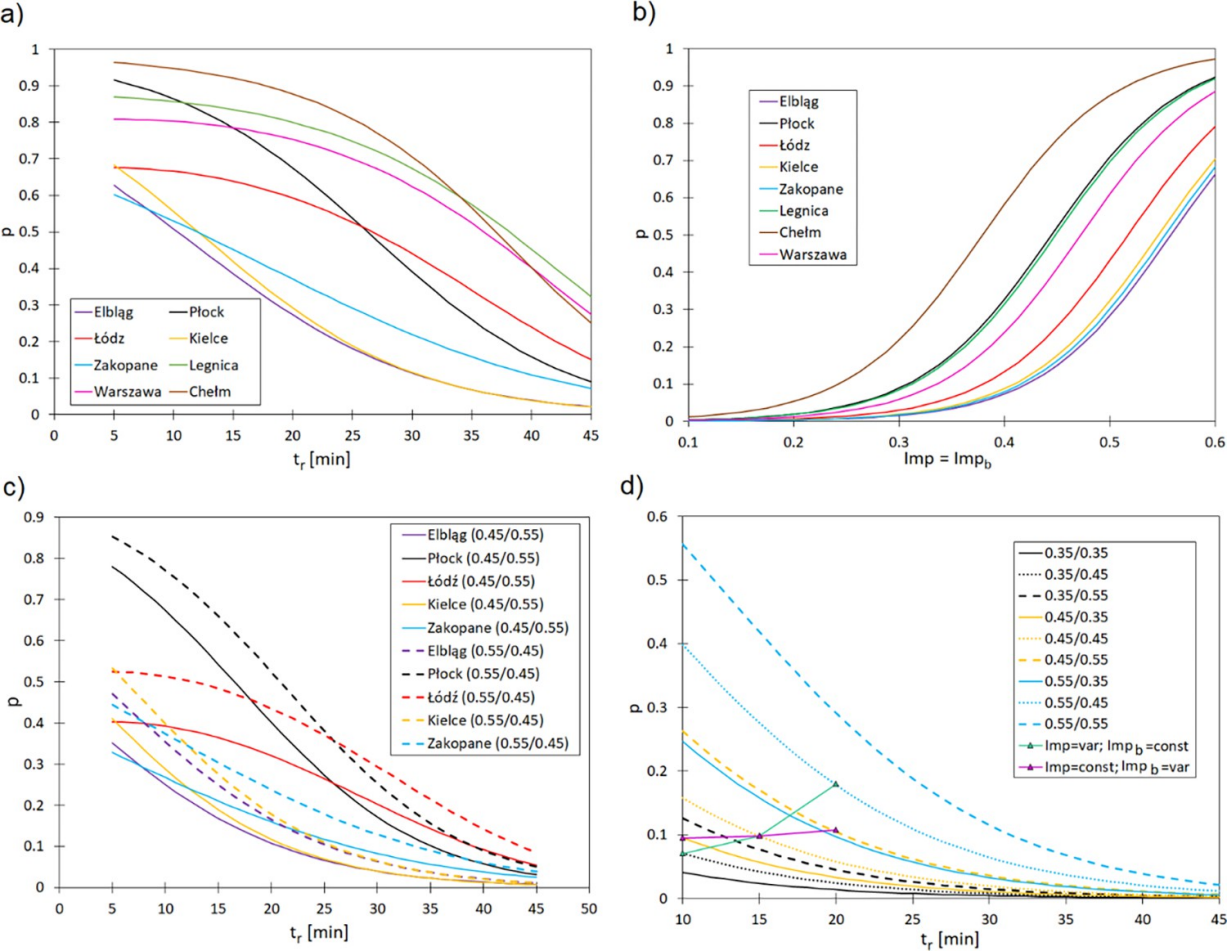
Influence of the convective rainfall event duration on the probability of stormwater flooding. (a) Influence of the convective rainfall event duration on the probability of stormwater flooding in small urban catchments (Imp = Imp_b_ = 0.55) in selected Polish cities. (b) Influence of the Imp = Imp_b_ values for t_r_ = 10 min on the probability of stormwater flooding in the selected Polish cities. (c) Influence of the rainfall duration and spatial distribution of the impervious area on the probability of stormwater flooding in the selected Polish cities. (d) Influence of the spatial distribution of the impervious area in a small catchment on the probability of stormwater flooding on the example of Kielce.

On the basis of the designated model for convective rainfall events related to their durations, reflecting the dependence of the maximum convective rainfall depth on the duration of rainfall events in Poland, the probability of stormwater flooding was calculated. This chapter describes five selected cities located along the meridian (on the longitude within a low range of variability) and within the main land relief belts: Elbląg (Baltic Sea coast), Płock (lakelands), Łódź (central lowlands), Kielce (uplands), and Zakopane (Carpathian Mountains). Three cities located in various parts of the central lowlands belt were additionally considered: Legnica in the southwest (Saxony-Lusatia Lowlands), Warsaw in the central part (Central Poland Lowlands), and Chełm in the southeast (Eastern Baltic-Belarus Lowland). By employing the developed logit model and using local rainfall models, the probability of stormwater flooding in each small urban catchment was determined for the assumed catchment characteristics; the calculation results are presented in Fig 5.

On the basis of the obtained p = f(t_r_) curves, it was noted that the highest p-values were obtained for Chełm, Płock, and Legnica, while the values were much lower for Kielce, Zakopane, and Elbląg (Fig 5A). While analyzing the determined curves, it emerges that stormwater flooding for the assumed imperviousness of the small urban catchment areas can occur at t_r_ < 20 min in Zakopane, whereas in Łódź and Płock, the t_r_ values are greater, at approximately 27 min. In Warsaw, Legnica, and Chełm, flooding can occur when rainfall events last 36–38 min. Additionally, the developed curves indicate the influence of the duration of a rainfall event on the stormwater flooding probability relation for the selected cities (Fig 5A and 5B). The significant influence of the local rainfall dynamics on stormwater flooding in the small urban catchments of the selected cities is also indicated by the determined p = f(Imp) curves for the rainfall event duration t_r_ = 10 min. In the case of Imp = Imp_b_ = 0.10–0.50, the highest p-values were obtained for Chełm, Płock, and Legnica, whereas the lowest p-values were obtained for Elbląg, Zakopane, and Kielce (the maximum difference between the calculated values p = f(Imp) is not greater than 3%).

The conducted analyses confirmed that the distribution of spatial imperviousness in urban areas has a significant impact on stormwater flooding in the selected regions of Poland. The highest stormwater flooding probability in an urban catchment within the analyzed cities was obtained when Imp > Imp_b_ (Fig 5C). Moreover, assuming p = 0.50, it can be stated that stormwater flooding in Imp < Imp_b_ catchments will occur for shorter convective rainfall events than in the case of Imp > Imp_b_.

The influence of the spatial distribution of an urban catchment imperviousness (e.g., in subsequent catchment urbanization phases) is presented in the example of Kielce (Fig 5D). Assuming Imp = 0.35 and t_r_ = 15 min, an increase in Imp_b_ from 0.35 to 0.55 raises the probability of stormwater flooding from 0.023 to 0.074, whilst analyzing the p = f(t_r_, Imp = 0.55, Imp_b_ = 0.35) and p = f(t_r_, Imp = 0.45, Imp_b_ = 0.55) curves, it can be observed that for the subsequent t_r_ values, the difference in the determined stormwater flooding probability does not exceed 0.026 (i.e. 7%). While comparing the dynamics of changes in catchment imperviousness, when p = f(t_r_, Imp = const = 0.45, Imp_b_ = var = 0.35–0.55) and p = f(t_r_, Imp = var = 0.35–0.55, Imp_b_ = const = 0.45), it can be stated that, in the second case, the calculated p-values were greater those calculated in the first case. An increase in the Imp value included in the range 0.35–0.55 for Imp_b_ = const raises the p-value from 0.071 to 0.180 (255%), whereas an increase in Imp_b_ = 0.35–0.55 for Imp = const raises the p-value from 0.096 to 0.108 (12%). Therefore, the obtained results indicate that in the sustainable development of urban catchments, location is a significant parameter; however, local rainfall dynamics should be properly taken into account as well. The significant impact of rainfall characteristics on the operation of objects located in the stormwater drainage network has been demonstrated by several authors.

De Paola and Ranucci [17], using long-term rainfall data from rain stations in Italy, showed the relationships between the rainfall characteristics, geographical locations of the studied stations, and the unit capacities of the studied tanks. The abovementioned results also correspond with the results of the calculations performed by Guo and Urbonas [70], as well as those of Guo and Cheng [71], who showed the impact of spatial variation in rainfall in the United States of America on the rainwater quality requiring treatment (due to high values of total suspended solids) and on the volume of reservoirs. In turn, to mitigate the potential stormwater flooding during the ongoing urbanization of catchments, it is recommended that imperviousness be decreased to reduce the probability of this unfavorable event. This can be achieved by assuming the appropriate catchment urbanization dynamics of the analyzed terrain and the area below the analyzed catchment.

### Influence of catchment features and local rainfall characteristics on the critical rainfall duration and intensity

**Fig 6:**
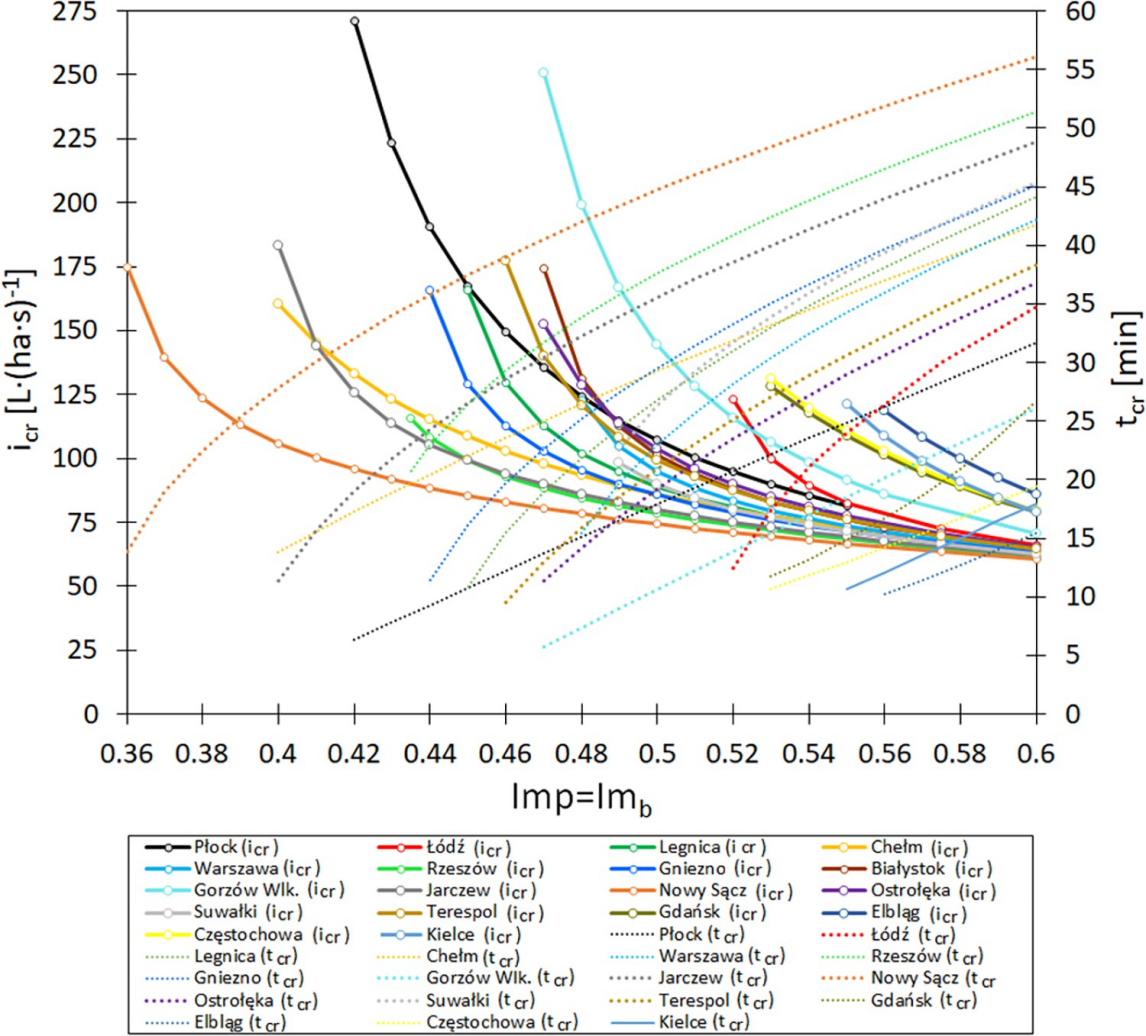
Influence of catchment imperviousness on the rainfall duration (t_cr_) and critical rainfall intensity (i_cr_) in selected Polish cities.

By applying a logit model to predict the probability of stormwater flooding in a small urban catchment described with Eq (14) and using the dependencies expressed in Eqs (5) and (6) to calculate the critical rainfall event durations, the influence of catchment features (Imp = Imp_b_) and local rainfall characteristics on the i_cr_ = f(Imp, ζ) (and, thus, t_cr_ = f(Imp, ζ) relations was analyzed. The analyses involved cities located in lakelands, central lowlands and mountains, by considering both their longitudes and latitudes, as well as their heights above sea level. Due to the vast surface area of the central lowlands, as well as the longitudinal and latitudinal extent of this region, the analyses were performed in six cities/towns (Białystok, Ostrołęka, Warsaw, Łódz, Jarczew, Legnica, and Chełm). In the case of the lakeland region, four cities were analyzed (Suwałki, Płock, Gniezno, and Gorzów Wielkopolski). In the simulations of the mountainous (Carpathian Mountains) and sub-mountainous regions, Rzeszów and Nowy Sącz were considered, and the uplands were represented by Kielce and Częstochowa; the Baltic Sea coast was represented by Gdańsk and Elbląg. The results of the conducted calculations are presented in Fig 6. The graph shows the curves describing the limit rainfall intensity, the exceeding of which determines the occurrence of wastewater discharge in the catchment depending on the impermeability of the area.

On the basis of the determined curves, it can be stated that the critical intensity of rainfall (i_cr_) resulting in stormwater flooding in a small urban catchment decreases as the impervious area increases. Hence, the critical rainfall duration (t_cr_) is extended. The obtained curves confirm the important influences of local rainfall characteristics on the dynamics of i_cr_ = f(Imp, ζ) and t_cr_ = f(Imp, ζ) changes.

Moreover, based on the determined curves, it can be stated that at different minimum Imp = Imp_b_ values in small urban catchments located in Polish cities, stormwater flooding may occur. The dependences determined in Fig 6 are complex, which is indicated by the different plots of the obtained curves, defined by the geographical location (latitude, longitude), topography, and height above sea level of each catchment. These features have significant effects on the local climate, which, combined with the characteristics of the small urban catchments, affect the occurrence of stormwater flooding.

The influence of the local climatic characteristics and imperviousness of the urban catchment areas on stormwater flooding has not been thoroughly analyzed. Therefore, it is difficult to relate these results to those obtained by other authors [14, 15, 66] because they were local. Other areas with spatial distributions of meteorological characteristics have not been subjected to analyses or simulations; thus, the influence of urban catchment characteristics on the operation of stormwater networks has not been established. During the sustainable development of urban catchments, the design of stormwater management systems should be based on regional rainfall models. This aspect is essential and has a significant influence on the operation of stormwater networks and their modernization. From the point of view of designing stormwater management systems, the questions of whether the implementation of a surface with greater permeability is sufficient or whether the application of adaptive solutions, e.g., blue-green infrastructure, aimed at managing stormwater at their intake location is required [72, 73] are vital. Equally important is the question which of the abovementioned solutions will be optimal and ensure the required hydraulic effect.

### Ranking of the areas sensitive to stormwater flooding for a small urban catchment

**Fig 7:**
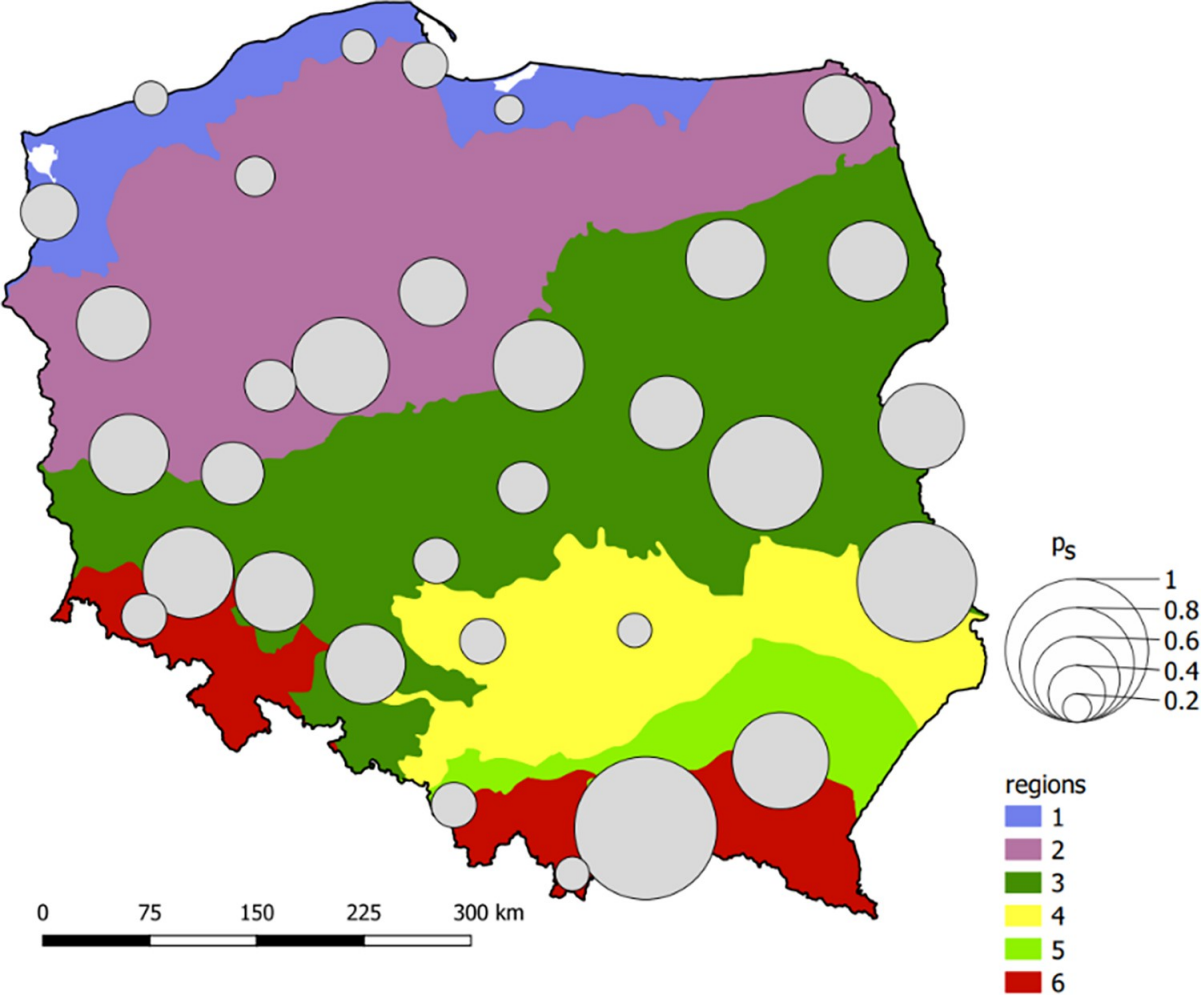
Variability in the stormwater flooding sensitivity index (p_s_) in Poland for selected cities and regions (region names are listed in Fig 1).

On the basis of the determined i_cr_ = f(Imp = Imp_b_) curves for the selected rainfall stations, as well as Eqs (8)–(10), sensitivity indices (p_s_) were determined (S2 and S3 Tables) presents the calculated case matrix J, J^’^, as described by Eqs (7), (8), and S4 Table presents the p_s_ values for 32 rainfall stations in Poland). Thus, the sensitivity of areas to stormwater flooding was established for the small urban catchments. Fig 7 was prepared based on the calculated p_s_ values and, thus, the determined ranking (from the most to least sensitive areas). High p_s_ values (>0.5) occur in the cities in eastern and southeastern Poland (Nowy Sącz, Chełm Lubelski, Jarczew, Rzeszów, Terespol, and Białystok), its central region (Gniezno and Płock) and the southwestern part of the central Silesia Lowland (Legnica, Wrocław, and Opole). These are the most thermally active areas. They are characterized by very high-intensity values of short rainfall events (>1 mm min^-1^).

In southeastern Poland, the rankings reflect the ascendance of continental climate features, which manifest themselves in summer by the increased thermal activity of the terrestrial surface. The highest total solar radiation values, exceeding 1800 MJ m^-2^ year^-1^ on average in the summer season (VI−VIII), are noted in this location [74]. Central Poland is an area with high absorbed solar radiation values, exceeding the mean daily values of 15 MJ m^-2^ (in summer). The share of energy spent for evaporation related to the net radiation balance in the area of Płock is exceptionally high, reaching over 70% [75]. The terrain features, especially the high differentiation of albedo (simultaneously unforested and lake areas), are conducive to free thermal convection. In turn, the Silesian Lowland is the region with the highest number of very hot days (T_max_ > 30°C), which is conducive to the development of free convection [50]. This area overlaps the region characterized by the longest period of intense rainfall events [76].

The cities located at the Baltic Sea coast (Elbląg, Lębork, Kołobrzeg, and Gdańsk), mountainous areas (Zakopane, Wisła, and Jelenia Góra) and uplands (Kielce and Częstochowa) occupy the far end of the ranking (p_s_ < 0.35). These are the areas with the lowest mean air temperature values in Poland and are characterized by high relative air humidity [50]. Low maximum intensity values of the shortest rainfall events, reaching less than 0.8 mm min^-1^ in the 1–10 min interval and 0.5 mm min^-1^ in the 11–20 min interval, occur in these regions. The low intensity values are also the effect of ‘ventilation’ in mountainous areas, which hinders the intensive development of free convection processes and the occurrence of short intensive rainfall events. In turn, relatively low intensities of short rainfall events in the cities located in northern Poland (coast) result from the influence of the Baltic Sea on the weakening of convective processes. The middle of the ranking corresponds to the cities mainly located in the central lowlands and lakelands. This region can be interpreted as a transitional area, where the features are partially associated with the top of the ranking (e.g., Ostrołęka, Toruń, and Suwałki have high maximum intensity values of short rainfall events), as well as to its end (Szczecin, Gdańsk have low maximum intensity values of short rainfall events).

### Aspects of sustainable development of small urban catchments in Poland

**Fig 8:**
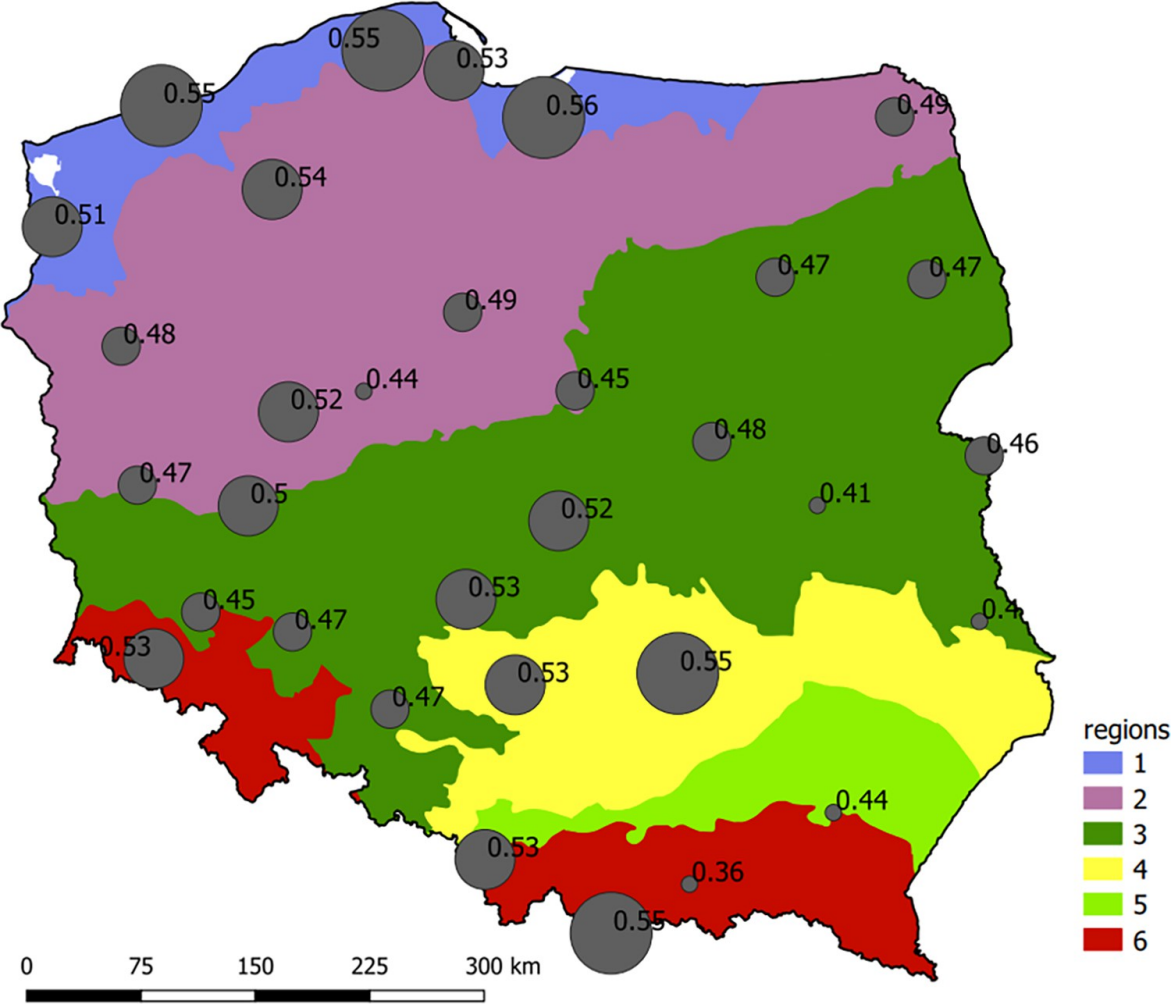
Maximum catchment imperviousness (Imp_gr_) for the analyzed rainfall stations, the exceedance of which results in stormwater flooding in the small urban catchments (region names are listed in Fig 1).

On the basis of Eqs (12, 13, 14) as well as the convective rainfall prediction models P_max_ = f(t_r_), the maximum Imp_gr_ value, the exceedance of which results in stormwater flooding in a small urban catchment, was determined for the analyzed rainfall stations. The obtained calculation results are presented in Fig 8. According to the illustrated Imp_gr_ variability in Poland, it can be stated that from the physical-geographic and meteorological viewpoints, the greatest possibilities of improving imperviousness (in terms of stormwater flooding) correspond, in the majority of cases, to the cities located in the northern part of the country (Baltic Sea coast).

From the analysis of the obtained results, it was noted that the issues with stormwater flooding in small urban catchments may occur when Imp_gr_ ≥ 0.50 in central lowland areas (Wieluń and Łódź), lakelands (Leszno and Poznań), uplands (Kielce and Częstochowa), and mountains (Wisła, Zakopane, and Jelenia Góra). In the mountains, stormwater flooding occurs in the small urban catchments with Imp_gr_ = 0.36 (Nowy Sącz) and in the case of the sub-mountainous basins, stormwater flooding occurs when Imp_gr_ = 0.44 (Rzeszów). In the central lowlands, the maximum imperviousness values differ greatly. The lowest Imp_gr_ was observed in the southeast part of the central lowlands. In turn, the calculated Imp_gr_ values in the middle part of the central lowlands are greater. The obtained calculation results confirm the fact that the development, urbanization, expansion, and revitalization of urban catchments should be sustainable, which minimizes the issues connected to the operation of stormwater networks.

## Advantages and disadvantages of the model

The proposed logit model may not work under the conditions of another city, with a different area size or topology, which will exhibit different retention characteristics, and lack accurate sewer network data. A small catchment size was used in this study, which allowed for eliminating some factors and simplifying the model. The developed models were verified for small areas. In order to analyze larger areas, it would be necessary to perform studies in other catchments, not necessarily in other cities. It is important that additional measurements be made under different water and soil conditions, different imperviousness, retention or sewer density. In order to eliminate the limitations of the model resulting from the variability of independent variables as well as rainfall depth, size of the catchment, channel volume, and density of the sewer network, it would be necessary to carry out studies in other catchments with different characteristics. This would allow verifying the model and the research methodology applied. Moreover, it would make the model universal.

The study sought a tool for identifying stormwater flooding in a catchment based on basic catchment characteristics, which in most cases are independent. The authors are aware of the limitations of the logit model, so appropriate simplifications were made in the analyses. Therefore, further analyses and modifications of the model will be needed to take into account factors not yet considered, which will allow broadening the applicability of the presented method. The areas differing from that described in the paper in terms of type and surface area, retention, imperviousness or density of water supply network will be analyzed in further studies.

## Conclusions

The analysis of stormwater flooding in urban catchments is extremely important when considering the modernization of the stormwater network in a catchment. The conducted calculations indicated that stormwater flooding in small catchments can be modeled using logistic regression based on rainfall data (rainfall depth and duration) and the catchment characteristics (imperviousness of the area and its spatial distribution, density of the stormwater network). The determined model can be implemented in other catchments, which is confirmed by the results of the simulations performed with the hydrodynamic model. On the basis of the determined logit model, an innovative analytic relationship was determined for calculating the maximum rainfall duration (of convective rainfall events) leading to stormwater overflows in small urban catchments, by considering the catchment characteristics as well as the local climate and geographical conditions in Poland. On the basis of the performed calculations, the maximum permissible imperviousness values were determined for small urban catchments in selected cities and regions of Poland, the exceedance of which may lead to stormwater overflows resulting from convective rainfall.

The proposed sensitivity index enabled the identification of small urban catchments in Poland, which are especially sensitive to stormwater flooding. The closer the value of the sensitivity index is to 1, the greater the sensitivity of the area to stormwater flooding is. In turn, the lower the sensitivity index value is, the lower the risk of stormwater flooding is. The performed calculations indicate that for the analyzed stations, the highest sensitivity index was obtained for Nowy Sącz, whereas the lowest was found for Elbląg, equal to 0.20. This is important when making appropriate decisions in terms of long- and short-term land development. In the long term, the obtained results enable the implementation of sustainable development plans for urban areas both locally and spatially, as demonstrated in the paper.

Although the presented methodology was shown using Poland as an example, the proposed methodology can be used for any country or climatic and geographical region. The basic condition is the variability of rainfall for the analyzed area. Considering the obtained calculation results, the determined logit model should be verified for other urban catchments (with various physical-geographic characteristics), and the number of rainfall stations should be increased to perform the regionalization of Poland in terms of the areas where small urban catchments are at risk of stormwater flooding. This methodology may turn out to help determining the strategies for managing stormwater in terms of the current legal regulations and branch solutions.

## Supporting information

S1 Table Geographical and rainfall characteristics for selected rainfall stations in Poland. (PDF)

S2 Table Matrix J. (PDF)

S3 Table Matrix J’. (PDF)

S4 Table Values of sensitivity indexes (p_s_) for selected precipitation stations in Poland. (PDF)

S5 Table Regression statistics. (PDF)

S1 File List of symbols and abbreviations. (PDF)
