# Supplementary material for: Flood occurrence analysis in small urban catchments in the context of regional variability
Source: PLoS One. 2022 Nov 3;17(11):e0276312. doi: 10.1371/journal.pone.0276312 (PMC9632778; doi:10.1371/journal.pone.0276312)
Supplement: S1 Table — (PDF) [file pone.0276312.s001.pdf]

Supporting information

Flood occurrence analysis in small urban catchments in the context of regional variability

S1 Table. Geographical and rainfall characteristics for selected rainfall stations in Poland

No.	Station name	Geographic coordinates (DD)		Altitude (m a.s.l.)	Precipitation total * (mm)	Coefficients values in equation (1)		
		Latitude	Longitude			a1	a2	a0
1	Białystok	53.11667	23.16667	148	576	-0.0026	0.3058	7.12
2	Chełm Lubelski	51.11667	23.50000	220	551	-0.0016	0.1868	11.0
3	Częstochowa	50.81667	19.10000	293	634	-0.0003	0.1153	7.18
4	Elbląg	54.16667	19.43333	38	690	-0.0005	0.1373	5.95
5	Gdańsk	54.38333	18.46667	137	541	-0.0015	0.1865	6.60
6	Gniezno	52.55000	17.56667	124	512	-0.0028	0.328	7.94
7	Gorzów Wlkp.	52.75000	15.28333	72	542	-0.0006	0.1387	7.84
8	Jarczew	51.81667	21.98333	182	560	-0.0028	0.3187	9.27
9	Jelenia Góra	50.90000	15.80000	342	689	-0.0007	0.1648	6.69
10	Kielce	50.81667	20.70000	260	618	-0.0003	0.1113	6.55
11	Kołobrzeg	54.18333	15.58333	3	609	-0.0006	0.1368	6.40
12	Legnica	51.21667	16.16667	122	525	-0.0024	0.3102	7.76
13	Leszno	51.83333	16.53333	91	545	-0.0032	0.3668	5.61
14	Lębork	54.55000	17.75000	17	700	-0.0007	0.1665	6.01
15	Łódź	51.73333	19.40000	184	569	-0.0029	0.3407	5.40
16	Nowy Sącz	49.61667	20.70000	292	731	-0.0030	0.3537	10.2
17	Opole	50.66667	17.96667	176	603	-0.0028	0.3392	7.07
18	Ostrołęka	53.08333	21.56667	95	541	-0.0022	0.2662	7.65
19	Płock	52.53333	19.66667	63	518	-0.0005	0.1152	9.63
20	Poznań	52.41667	16.83333	86	523	-0.0029	0.3407	5.30
21	Rzeszów	50.10000	22.05000	200	653	-0.0034	0.3953	7.67
22	Suwałki	54.13333	22.95000	184	598	-0.0034	0.4013	5.99
23	Szczecin	53.40000	14.61667	1	557	-0.0013	0.1535	7.48
24	Szczecinek	53.71667	16.68333	137	618	-0.0010	0.2233	5.74
25	Terespol	52.06667	23.61667	133	520	-0.0024	0.2792	7.67
26	Toruń	53.05000	18.58333	69	537	-0.0025	0.2962	6.66
27	Warszawa	52.28333	20.98333	98	531	-0.0029	0.3458	6.72
28	Wieluń	51.21667	18.58333	195	590	-0.0003	0.1138	5.45
29	Wisła	49.65000	18.98333	685	1176	-0.0007	0.1825	6.17
30	Wrocław	51.10000	16.88333	120	537	-0.0027	0.3235	7.07
31	Zakopane	49.30000	19.95000	857	1017	-0.0009	0.2160	5.36
32	Zielona Góra	51.93333	15.53333	192	584	-0.0025	0.2937	7.78

*- mean total annual precipitation in period 1981-2010
