# Supplementary material for: Flood occurrence analysis in small urban catchments in the context of regional variability
Source: PLoS One. 2022 Nov 3;17(11):e0276312. doi: 10.1371/journal.pone.0276312 (PMC9632778; doi:10.1371/journal.pone.0276312)
Supplement: S2 Table — (PDF) [file pone.0276312.s002.pdf]

Supporting information

Flood occurrence analysis in small urban catchments in the context of regional variability

S2 Table. Matrix J

i/k	Imp		Białystok	Chełm Lubelski	Częstochowa	Elbląg	Gdańsk	Gniezno	Gorzów Wlkp.	Jarczew	Jelenia Góra	Kielce	Kołobrzeg	Legnica	Leszno	Lębork	Lublin	Łódź	Nowy Sącz	Opole	Ostrołęka	Płock	Poznań	Rzeszów	Suwałki	Szczecin	Szczecinek	Terespol	Toruń	Warszawa	Wieluń	Wisła	Wrocław	Zakopane	Zielona Góra
1	0.36		0.00	0.00	0.00	0.00	0.00	0.00	0.00	0.00	0.00	0.00	0.00	0.00	0.00	0.00	0.00	0.00	174.73	0.00	0.00	0.00	0.00	0.00	0.00	0.00	0.00	0.00	0.00	0.00	0.00	0.00	0.00	0.00	0.00
2	0.37		0.00	0.00	0.00	0.00	0.00	0.00	0.00	0.00	0.00	0.00	0.00	0.00	0.00	0.00	0.00	0.00	139.34	0.00	0.00	0.00	0.00	0.00	0.00	0.00	0.00	0.00	0.00	0.00	0.00	0.00	0.00	0.00	0.00
3	0.38		0.00	0.00	0.00	0.00	0.00	0.00	0.00	0.00	0.00	0.00	0.00	0.00	0.00	0.00	0.00	0.00	123.40	0.00	0.00	0.00	0.00	0.00	0.00	0.00	0.00	0.00	0.00	0.00	0.00	0.00	0.00	0.00	0.00
4	0.39		0.00	0.00	0.00	0.00	0.00	0.00	0.00	0.00	0.00	0.00	0.00	0.00	0.00	0.00	0.00	0.00	113.31	0.00	0.00	0.00	0.00	0.00	0.00	0.00	0.00	0.00	0.00	0.00	0.00	0.00	0.00	0.00	0.00
5	0.4		0.00	160.49	0.00	0.00	0.00	0.00	0.00	0.00	0.00	0.00	0.00	0.00	0.00	0.00	0.00	0.00	106.02	0.00	0.00	0.00	0.00	0.00	0.00	0.00	0.00	0.00	0.00	0.00	0.00	0.00	0.00	0.00	0.00
6	0.41		0.00	145.10	0.00	0.00	0.00	0.00	0.00	144.17	0.00	0.00	0.00	0.00	0.00	0.00	0.00	0.00	100.34	0.00	0.00	0.00	0.00	0.00	0.00	0.00	0.00	0.00	0.00	0.00	0.00	0.00	0.00	0.00	0.00
7	0.42		0.00	133.06	0.00	0.00	0.00	0.00	0.00	125.52	0.00	0.00	0.00	0.00	0.00	0.00	0.00	0.00	95.70	0.00	0.00	0.00	0.00	0.00	0.00	0.00	0.00	0.00	0.00	0.00	0.00	0.00	0.00	0.00	0.00
8	0.43		0.00	123.36	0.00	0.00	0.00	0.00	0.00	113.82	0.00	0.00	0.00	0.00	0.00	0.00	0.00	0.00	91.79	0.00	0.00	0.00	0.00	0.00	0.00	0.00	0.00	0.00	0.00	0.00	0.00	0.00	0.00	0.00	0.00
9	0.44		0.00	115.35	0.00	0.00	0.00	165.64	0.00	105.48	0.00	0.00	0.00	0.00	0.00	0.00	0.00	0.00	88.42	0.00	0.00	0.00	0.00	108.47	0.00	0.00	0.00	0.00	0.00	0.00	0.00	0.00	0.00	0.00	0.00
10	0.45		0.00	108.60	0.00	0.00	0.00	128.88	0.00	99.09	0.00	0.00	0.00	166.02	0.00	0.00	0.00	0.00	85.45	0.00	0.00	167.21	0.00	99.14	0.00	0.00	0.00	0.00	0.00	0.00	0.00	0.00	0.00	0.00	0.00
11	0.46		0.00	102.82	0.00	0.00	0.00	112.64	0.00	93.95	0.00	0.00	0.00	129.52	0.00	0.00	0.00	0.00	82.81	0.00	0.00	149.39	0.00	92.92	0.00	0.00	0.00	177.09	0.00	0.00	0.00	0.00	0.00	0.00	0.00
12	0.47		174.40	97.80	0.00	0.00	0.00	102.63	0.00	89.67	0.00	0.00	0.00	112.54	0.00	0.00	0.00	0.00	80.42	129.17	152.46	135.42	0.00	88.23	0.00	0.00	0.00	139.95	0.00	0.00	0.00	0.00	151.31	0.00	146.87
13	0.48	J =	131.27	93.39	0.00	0.00	0.00	95.52	199.19	86.02	0.00	0.00	0.00	102.04	0.00	0.00	0.00	0.00	78.24	109.29	128.54	124.16	0.00	84.44	0.00	0.00	0.00	120.54	0.00	122.54	0.00	0.00	119.81	0.00	124.01
14	0.49		112.59	89.49	0.00	0.00	0.00	90.07	166.79	82.84	0.00	0.00	0.00	94.64	0.00	0.00	0.00	0.00	76.23	98.65	113.78	114.89	0.00	81.27	98.26	0.00	0.00	108.17	151.91	104.74	0.00	0.00	105.28	0.00	110.45
15	0.5		101.39	85.99	0.00	0.00	0.00	85.68	144.53	80.02	0.00	0.00	0.00	89.01	168.99	0.00	0.00	0.00	74.38	91.51	103.55	107.12	0.00	78.52	89.75	0.00	0.00	99.40	121.45	95.09	0.00	0.00	96.19	0.00	101.17
16	0.51		93.61	82.83	0.00	0.00	0.00	82.00	128.28	77.50	0.00	0.00	0.00	84.50	107.09	0.00	0.00	0.00	72.65	86.20	95.92	100.51	0.00	76.10	84.26	138.44	0.00	92.74	105.99	88.55	0.00	0.00	89.71	0.00	94.25
17	0.52		87.75	79.97	0.00	0.00	0.00	78.85	115.88	75.21	0.00	0.00	0.00	80.75	93.40	0.00	0.00	123.31	71.03	81.98	89.94	94.81	144.30	73.93	80.15	123.21	0.00	87.43	96.17	83.63	0.00	0.00	84.71	0.00	88.81
18	0.53		83.10	77.35	130.92	0.00	128.23	76.10	106.09	73.13	128.55	0.00	0.00	77.56	85.91	0.00	0.00	99.88	69.51	78.48	85.06	89.84	104.49	71.96	76.85	111.61	0.00	83.06	89.15	79.70	143.13	148.36	80.66	0.00	84.36
19	0.54		79.25	74.95	119.87	0.00	117.75	73.65	98.16	71.21	113.53	0.00	0.00	74.78	80.72	0.00	0.00	89.22	68.08	75.50	80.99	85.48	91.65	70.16	74.07	102.46	126.17	79.36	83.77	76.43	126.86	124.83	77.27	0.00	80.61
20	0.55		75.98	72.73	110.55	0.00	108.86	71.45	91.60	69.43	102.40	121.11	118.12	72.33	76.75	121.16	0.00	82.59	66.72	72.91	77.50	81.61	84.12	68.49	71.67	95.03	107.35	76.16	79.45	73.63	114.42	109.18	74.36	130.22	77.39
21	0.56		73.15	70.67	102.57	118.69	101.21	69.44	86.07	67.77	93.81	108.56	105.78	70.13	73.53	110.95	112.06	77.78	65.43	70.61	74.46	78.15	78.84	66.93	69.54	88.86	95.19	73.36	75.86	67.99	104.54	98.00	71.81	108.90	74.56
22	0.57		70.65	68.75	95.66	108.36	94.57	67.61	81.34	66.22	86.96	98.74	96.25	68.14	70.80	102.33	97.21	74.01	64.20	68.54	71.77	75.04	74.79	65.48	67.63	83.66	86.62	70.87	72.79	69.00	96.49	89.58	69.54	95.53	69.79
23	0.58		68.42	66.96	89.63	99.68	88.74	65.91	77.25	64.76	81.36	90.84	88.67	66.32	68.44	94.95	87.27	70.91	63.02	66.66	69.38	72.22	71.50	64.11	65.89	79.19	80.21	68.63	70.12	67.03	89.78	83.00	67.49	86.30	67.74
24	0.59		66.40	65.28	84.31	92.29	83.60	64.34	73.67	63.38	76.69	84.35	82.49	64.65	66.35	88.57	80.11	68.27	61.89	64.94	67.21	69.66	68.74	62.82	64.28	75.30	75.20	66.60	67.76	65.24	84.07	77.69	65.63	79.50	65.87
25	0.6		64.56	63.70	79.59	85.91	79.01	62.86	70.51	62.07	72.73	78.92	77.34	63.10	64.47	82.98	74.66	65.98	60.81	63.35	65.24	67.32	66.34	61.59	62.80	71.89	71.16	64.73	65.64	63.59	79.15	73.32	63.92	74.26	64.14
