# Supplementary material for: Flood occurrence analysis in small urban catchments in the context of regional variability
Source: PLoS One. 2022 Nov 3;17(11):e0276312. doi: 10.1371/journal.pone.0276312 (PMC9632778; doi:10.1371/journal.pone.0276312)
Supplement: S4 Table — (PDF) [file pone.0276312.s004.pdf]

Supporting information

Flood occurrence analysis in small urban catchments in the context of regional variability

**S4 Table. Values of sensitivity indexes ($p_s$) for selected precipitation stations in Poland**

| Cities | $p_s$ |
|---|---|
| Nowy Sącz | 1.00 |
| Chełm Lubelski | 0.84 |
| Jarczew | 0.80 |
| Gniezno | 0.68 |
| Rzeszów | 0.68 |
| Legnica | 0.64 |
| Płock | 0.64 |
| Terespol | 0.60 |
| Białystok | 0.56 |
| Lublin | 0.56 |
| Opole | 0.56 |
| Ostrołęka | 0.56 |
| Wrocław | 0.56 |
| Zielona Góra | 0.56 |
| Gorzów Wlkp. | 0.52 |
| Warszawa | 0.52 |
| Suwałki | 0.48 |
| Toruń | 0.48 |
| Leszno | 0.44 |
| Szczecin | 0.40 |
| Łódź | 0.36 |
| Poznań | 0.36 |
| Częstochowa | 0.32 |
| Gdańsk | 0.32 |
| Jelenia Góra | 0.32 |
| Wieluń | 0.32 |
| Wisła | 0.32 |
| Szczecinek | 0.28 |
| Kielce | 0.24 |
| Kołobrzeg | 0.24 |
| Lębork | 0.24 |
| Zakopane | 0.24 |
| Elbląg | 0.20 |
