# Supplementary material for: Flood occurrence analysis in small urban catchments in the context of regional variability
Source: PLoS One. 2022 Nov 3;17(11):e0276312. doi: 10.1371/journal.pone.0276312 (PMC9632778; doi:10.1371/journal.pone.0276312)
Supplement: S5 Table — (PDF) [file pone.0276312.s005.pdf]

Supporting information

Flood occurrence analysis in small urban catchments in the context of regional variability

S5 Table. Regression statistics

Independent variable	Empirical coefficients (α_q)	p – test
P_{tot}	0.483	0.0023
t_r	-0.176	0.0032
Imp	9.899	0.0231
Impu	5.317	0.0312
Gk	40.441	0.0125
Intercept	-12.488	0.0219
Sens = 95.29%		
Spec = 92.23%		
Acc = 93.96%		
