# Supplementary material for: Flood occurrence analysis in small urban catchments in the context of regional variability
Source: PLoS One. 2022 Nov 3;17(11):e0276312. doi: 10.1371/journal.pone.0276312 (PMC9632778; doi:10.1371/journal.pone.0276312)
Supplement: S1 File — (PDF) [file pone.0276312.s006.pdf]

Supporting information

Flood occurrence analysis in small urban catchments in the context of regional variability

S1 File. List of symbols and abbreviations

a_1, a_2, a_0 – coefficients estimated in a polynomial describing the relationship $P_{max} = f(t_r, \zeta)$ (-),

dH – maximum height difference (in m) of the land coordinates in the catchment,

F – catchment area (ha),

G_k – density of the stormwater network, defined as the length of the main collector via the impervious surface of the catchment area,

h_{alt} – the land ordinate measured from sea level (m a.s.l.),

Imp – imperviousness of the area (-),

Imp_b – imperviousness of the areas located below the analyzed catchment (-),

Imp_{gr} – maximum impervious area, the exceedance of which leads to stormwater flooding (-),

i_{cr} – minimum average (convective) rainfall intensity leading to stormwater flooding in a given small urban catchment area (L s^{-1} ha^{-1}),

k – number of regions (rainfall stations) considered for calculation,

L_k – length of the collector measured from point $x_{A,B,C,D}$ to the diversion chamber DC (m),

L_{tot} – length of the main collector in the catchment,

P_a – annual (multiannual) rainfall for k at a given rainfall station (mm),

P_{max} – maximum depth of convective rainfall, calculated from the relationship $P_{max} = f(t_r, \zeta)$ (mm),

p – the probability of stormwater flooding in a rainfall event (-),

p_s – stormwater flooding sensitivity index (-),

SENS – sensitivity (%),

SPEC – specificity (%),

SWMM – storm water management model,

t_{cr} – maximum duration of (convective) rainfall leading to a stormwater flood event in a given small urban catchment area (min),

t_r – duration of convective rainfall (min),

x_{long} – longitude,

y_{lat} – latitude,

α_1, α_2, α_3, α_4 – coefficients estimated in the logistic regression model (-).
